# HIV-1 Integrase Inhibitors with Modifications That Affect Their Potencies against Drug Resistant Integrase Mutants

**DOI:** 10.1021/acsinfecdis.0c00819

**Published:** 2021-03-09

**Authors:** Steven J. Smith, Xue Zhi Zhao, Dario Oliveira Passos, Valerie E. Pye, Peter Cherepanov, Dmitry Lyumkis, Terrence R. Burke, Stephen H. Hughes

**Affiliations:** † HIV Dynamics and Replication Program, Center for Cancer Research, National Cancer Institute, Frederick, Maryland 21702, United States; ‡ Chemical Biology Laboratory, Center for Cancer Research, National Cancer Institute, Frederick, Maryland 21702, United States; § Laboratory of Genetics, The Salk Institute for Biological Studies, La Jolla, California 92037, United States; ∥ Chromatin Structure and Mobile DNA Laboratory, The Francis Crick Institute, London NW1 1AT, U.K.; ⊥ St Mary’s Hospital, Department of Infectious Disease, Imperial College London, Section of Virology, Norfolk Place, London W2 1PG, U.K.; # Department of Integrative Structural and Computational Biology, The Scripps Research Institute, La Jolla, California 92037, United States

**Keywords:** integrase, strand transfer, inhibition, potency, mutant, susceptibility

## Abstract

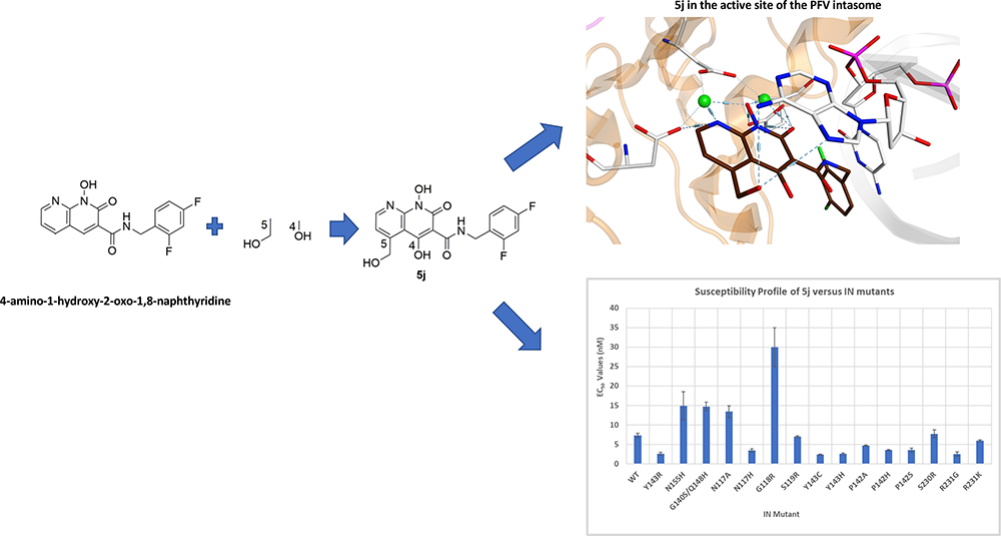

Integrase strand transfer inhibitors (INSTIs) block the integration step of the retroviral lifecycle and are first-line drugs used for the treatment of HIV-1/AIDS. INSTIs have a polycyclic core with heteroatom triads, chelate the metal ions at the active site, and have a halobenzyl group that interacts with viral DNA attached to the core by a flexible linker. The most broadly effective INSTIs inhibit both wild-type (WT) integrase (IN) and a variety of well-known mutants. However, because there are mutations that reduce the potency of all of the available INSTIs, new and better compounds are needed. Models based on recent structures of HIV-1 and red-capped mangabey SIV INs suggest modifications in the INSTI structures that could enhance interactions with the 3′-terminal adenosine of the viral DNA, which could improve performance against INSTI resistant mutants. We designed and tested a series of INSTIs having modifications to their naphthyridine scaffold. One of the new compounds retained good potency against an expanded panel of HIV-1 IN mutants that we tested. Our results suggest the possibility of designing inhibitors that combine the best features of the existing compounds, which could provide additional efficacy against known HIV-1 IN mutants.

Integrase strand transfer inhibitors (INSTIs), when used in combination with two reverse transcriptase inhibitors (RTIs), are the standard treatment for HIV-1 infections.^1^ INSTIs selectively block the strand transfer (ST) reaction, which is the second step catalyzed by the viral enzyme integrase (IN) (after 3′-processing). As the name implies, INSTIs prevent the insertion of viral DNA into the genome of the host cells.^2−4^ At present, there are five FDA-approved INSTIs, raltegravir (RAL), elvitegravir (EVG), dolutegravir (DTG), and bictegravir (BIC); cabotegravir (CAB) is licensed in Canada^5^ and has just been approved for use in the United States (Figure 1A). INSTIs specifically bind at the active site of IN when it is engaged with a viral DNA end. The generalized INSTI pharmacophore comprises two key elements: a metal chelating scaffold, optimized to bind a pair of Mg^2+^ ions in the IN active site, and a halobenzyl side chain, connected to the core by a flexible linker, which binds to viral DNA.^4^ All of the FDA-approved INSTIs potently inhibit the replication of wild-type (WT) HIV-1. However, it is relatively easy for HIV-1 to develop resistance to the first-generation INSTIs RAL and EVG, which share overlapping resistance profiles.^6−10^ Conversely, the second-generation INSTIs, DTG and BIC, retain good activity against common RAL- and EVG-resistant HIV-1 strains, and it appears to be more difficult for the virus to develop resistance to the second generation INSTIs.^11−19^ The ability of DTG and BIC to inhibit the replication of many of the RAL- and EVG-resistant mutants seems to be related to their extended tricyclic scaffolds.^19−21^

**Figure 1:**
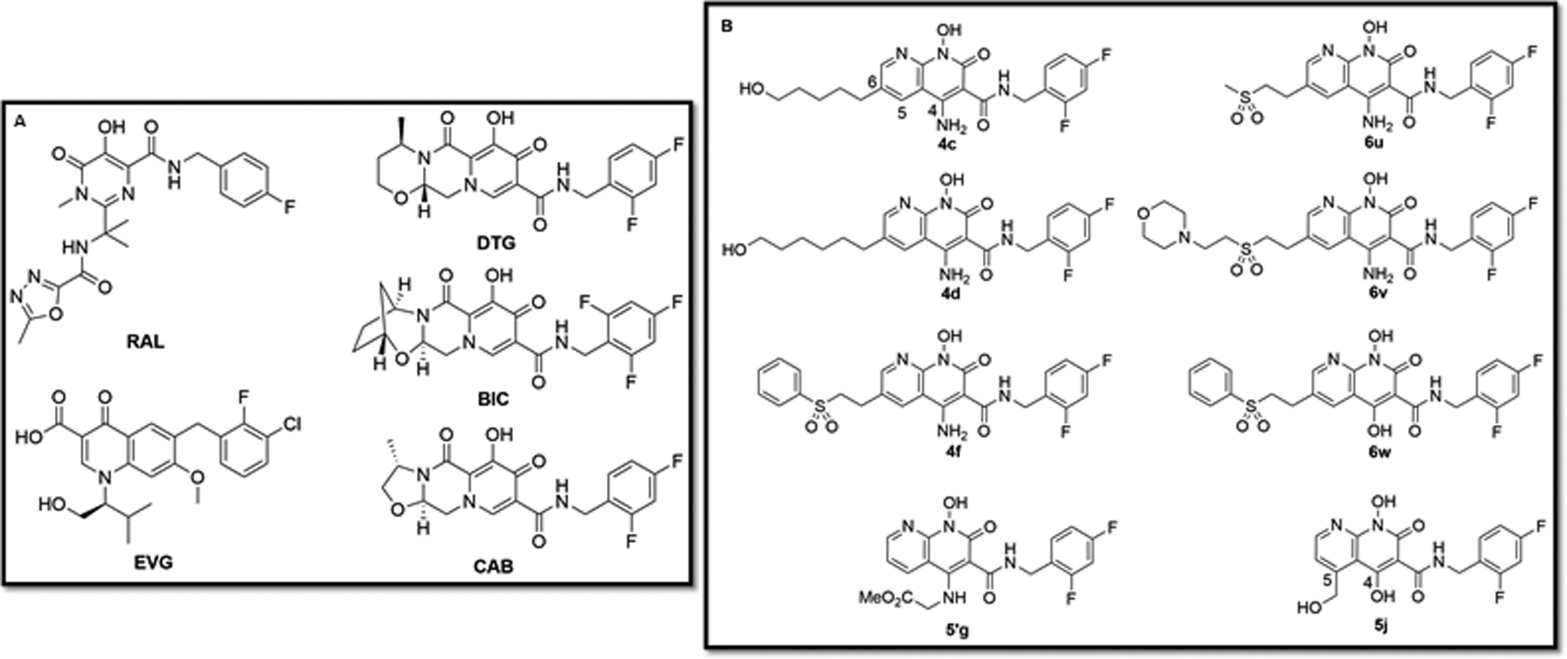
Chemical structures of the INSTIs. (A) The chemical structures of the clinically relevant INSTIs. (B) The chemical structures of our 4-amino-1-hydroxy-2-oxo-1,8-naphthyridine-containing compounds are shown.

INSTIs contain a central pharmacophore with electronegative atoms positioned to engage two Mg^2+^ cofactors in the IN active site. Metal ion engagement is a unifying feature shared by all INSTIs. Structural analyses, using red-capped mangabey SIV (SIVrcm) IN, revealed that the INSTI-resistant mutations G140S/Q148H affect INSTI binding by disrupting the secondary coordination shells of the Mg^2+^ ions in the IN active site.^21^ However, there are modifications to the INSTI scaffold that can compensate for this loss of binding affinity. For example, the presence of the oxazine ring in DTG and the oxazepine ring in BIC allow these compounds to make stabilizing interactions with backbone atoms of N117 and G118 in the IN β4-α2 loop, contributing to the improved activity of the second-generation INSTIs against the mutants.^21^ However, in recent clinical studies, INSTI-experienced patients who were switched to a salvage therapy regimen that included DTG showed reduced response rates to this drug.^22,23^ The poor response occurred in HIV-1 infected patients who had previously been treated with RAL and had acquired mutations in IN at positions G140 and Q148. Following a switch to DTG containing regimens, the viruses acquired additional mutations, primarily at positions L74, E92, or E138, resulting in a reduced susceptibility to DTG. Although BIC retained potency against these triple mutants, it was susceptible to other combinatorial mutations such as V72I/E138K/Q148K, G140S/Q148H/G149A, and the quadruple mutant L74M/G140S/S147G/Q148K.^24^ Furthermore, the majority of the INSTI-resistant triple mutants we tested caused a substantially decreased susceptibility to CAB.^19^ These observations underscore the pressing need to continue developing new INSTIs that are able to maintain potency against emerging mutations in HIV-1 IN, especially those that retain potency against the multimutation variants containing the G140S/Q148H double mutant.

We recently reported the development of a number of 4-amino-1-hydroxy-2-oxo-1,8-naphthyridine-containing INSTIs.^19,25,26^ Several of these compounds have modifications at the 6-position on the naphthyridine scaffold (Figure 1B). Most members of this series are potent inhibitors of WT HIV-1 and many well-characterized RAL-, EVG-, and DTG-resistant mutants. One compound in particular, **4d**, which has an extended hexanol modification at the 6-position, was better able to broadly inhibit a panel of INSTI-resistant triple mutants than either DTG or BIC (Figure 1B).^19^

Previous X-ray crystal structures of our INSTIs bound to the active site of the prototype foamy virus (PFV) IN in a complex with viral DNA (intasome) were instrumental in guiding our design and development of compounds that are broadly effective against IN mutants.^20,25−30^ However, recent cryo-EM structures of compounds **4c**, **4d**, and **4f** bound to HIV-1 intasomes revealed crucial differences in the binding of these compounds to the IN active sites in PFV and HIV-1 intasomes.^24^ The structures also showed that the binding of all of the broadly effective compounds to the active site of HIV-1 IN occurs within the substrate envelope. As was originally shown for protease inhibitors, the virus is less likely to develop resistance to inhibitors that stay within the normal substrate envelope.^31−33^ Similarly, HIV-1 IN is likely to have more difficulty discriminating between its natural DNA substrates and INSTIs that conform to the substrate envelope.^24,26^ In addition, because the recent structures of HIV-1 and SIVrcm intasomes are at high enough resolution to show ordered waters,^21,24^ it might be possible to develop INSTIs with modifications that mimic the interactions of the ordered waters (discussed below).

We synthesized new compounds that have modifications to the 4-, 5-, and 6-positions of the naphthyridine scaffold (Figure 1B). We measured the ability of DTG, **4d**, **4f**, and our new compounds to inhibit the replication of a panel of INSTI-resistant mutants. The panel includes additional IN mutants that have changes at amino acid positions that are predicted, by molecular modeling, to affect the binding of some of the compounds. We find that subtle changes in the structure of INSTIs can significantly affect their ability to inhibit INSTI-resistant mutants. We also explored the structure of these compounds in the context of HIV IN models and discuss how these modifications can be used in the design of next-generation INSTIs.

## Results

### Ligand Design and Synthesis

Previously, we reported two X-ray crystal structures of the PFV intasome with our 4-amino-1-hydroxy-2-oxo-1,8-naphthyridine-containing compounds bound at the active site, in which a molecule of the buffer 2-(*N*-morpholino)ethanesulfonic acid (MES) was found within the IN active site (PDB IDs: 5MMA and 5FRM).^25,26^ The sulfonic acid moiety of the MES molecule was bound in a pocket that was occupied by a phosphoryl linkage of the 3′-terminal dinucleotides within the PFV intasome when unprocessed viral DNA is bound for the 3′-processing reaction. The same pocket was occupied by target DNA in the PFV target DNA capture complex.^34^ Moreover, the buffer molecule was bound in a position similar to where the 6-substituents of our 4-amino-1-hydroxy-2-oxo-1,8-naphthyridine-containing compounds were bound to PFV IN.^25,26^ The positions where the 6-substituents in our 4-amino-1-hydroxy-2-oxo-1,8-naphthyridine-containing compounds interacted with PFV IN are similar to the position occupied by the terminal nucleotide of what will be the transferred strand in unprocessed viral DNA.^25,26^ Although there are important differences in the previously published PFV and the new HIV-1 and SIV_rcm_ intasome structures,^21,24^ the substituents at the 6-position of our compounds appear to interact with the same region as the ends of the unprocessed viral DNA. These new structures also showed that there are several ordered water molecules bound in and around the IN active site (Figure S1). In an attempt to target these bound waters, we used the bound MES buffer molecule in the PFV structures as a model to design a series of sulfone-containing analogs **6u**, **6v**, and **6w**. The sulfone-containing substituents were also intended to mimic the extension of unprocessed viral DNA end (Figure 1). The analysis included a previously prepared compound, **5′g**, which has methyl glycinate group appended to the 4-amino group of the naphthyridine scaffold and another analogue having a hydroxyl group at the 4-position with a hydroxymethyl at the 5-position (**5j**). These modifications were made to determine whether other hydrophilic modifications would help the new compounds retain efficacy against the current panel of mutants.

### Antiviral Activities of the New Compounds against RAL-Resistant Mutants

**Figure 2:**
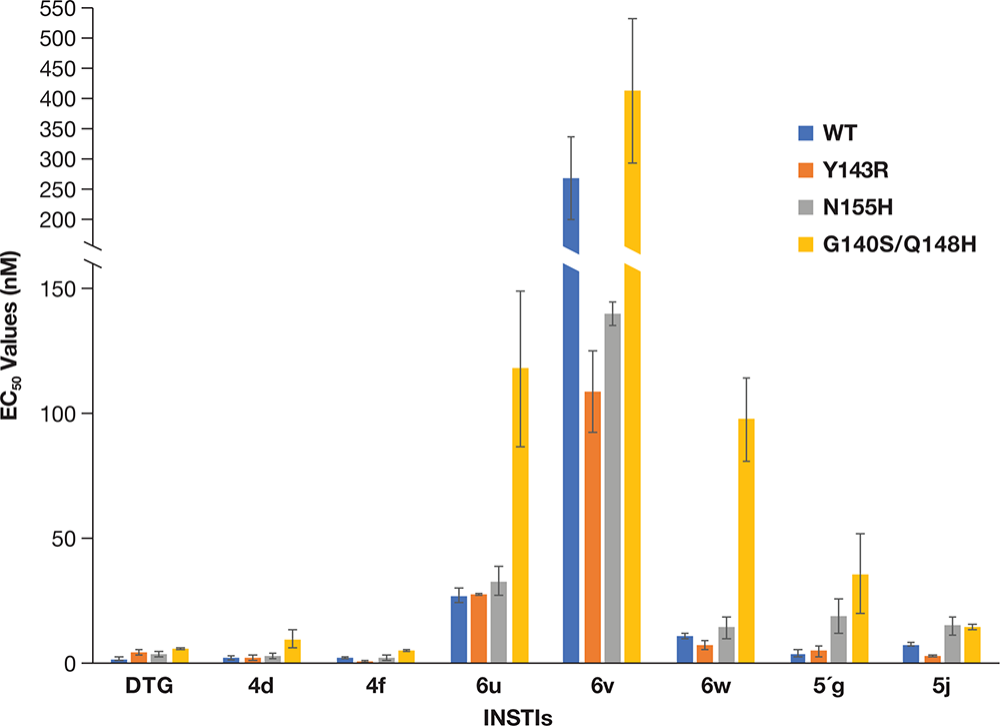
Antiviral activities of the new compounds against RAL-resistant mutants. The EC_50_ values were determined using a vector that carries WT HIV-1 IN and the RAL-resistant mutants in a single round infection assay. The potencies of DTG, **4d**, **4f**, and **5′g** have been previously reported.^25,26^ The previously reported data are shown to simplify comparisons with the data for the new compounds **6u**, **6v**, **6w**, and **5j**. To better illustrate the higher EC_50_ values, the *y*-axis was broken between 150 and 200 nM. Error bars represent the standard deviations of independent experiments, *n* = 4, performed in triplicate. The graph has a maximum value of 550 nM.

We previously reported the antiviral activities of DTG, **4d**, and **4f** against several well-characterized RAL-resistant mutants.^20,25,26^ In particular, we showed that **4f** potently inhibits the Y143R, N155H, and the G140S/Q148H RAL-resistant mutants (≤5.0 nM; all fold changes [FCs] ≤ 2.6). Similar results were obtained with DTG and **4d**. In the current work, we examined **6u**, **6v**, and **6w**, which are new INSTIs that have structures that are closely related to **4f**, except that the sulfonylphenyl group has been replaced (Figure 2; Table S1A). Compound **6u** has a methylsulfonyl-containing substituent at the 6-position; **6v** has an extended morpholinoethylsulfonyl group, which we designed to mimic the MES (morpholinoethylsulfonic acid) buffer molecule in our PFV IN cocrystal structures, and **6w**, which has the same 6-tethered phenylsulfonyl-containing substituent as **4f** (Figure 1). We replaced the 4-amino group of the naphthyridine scaffold of **4f** with a 4-hydroxyl group in **6w**. Based on our antiviral results, replacing the phenylsulfonyl group of **4f** with the smaller methylsulfonyl group (**6u**) resulted in a loss of potency against WT HIV-1 (27.0 ± 3.2 nM) compared to **4f** (2.0 ± 0.1 nM) (Figure 2, Table S1A). Substituting the phenyl group of **4f** with a more extended and bulkier morpholinoethylsulfonyl group (**6v**) caused a dramatic loss in potency against WT HIV-1 (267.9 ± 68.8 nM). Replacing a hydroxyl group for the 4-amino group of **4f** to give **6w** caused a modest decrease in potency against WT HIV-1 (10.6 ± 1.0 nM).

To compare the effectiveness of these new INSTIs to the FDA-approved INSTIs and a selection of our previously studied INSTIs, we compared the EC_50_ values and calculated the fold change (FC) in the EC_50_ values for the IN mutants versus WT HIV-1. In testing the new INSTIs, identifying the proper biological cutoffs would be helpful. However, because we do not know whether or not an INSTI that is in preclinical testing would be effective at inhibiting the INSTI-resistant mutants if the compound were to be used clinically, the Monogram FC cutoffs for resistance for DTG were used as a guide.^35^ Based on the DTG cutoffs, if the FCs are below 4, we assumed that the compound is likely to retain inhibitory efficacy. Conversely, if the FCs exceed 13, there is likely to be resistance. Finally, if the FCs are between 4 and 13, the virus may retain partial sensitivity to the compound. To facilitate the comparisons of the compounds, the ratios of the FCs for our compounds for WT IN and the various mutants were compared to the FCs for DTG against WT IN and the IN mutants. However, some of the new compounds that have small FCs are weakly potent against WT and the IN mutants, which means the FCs alone are not sufficient to judge the potential usefulness of the new compounds. For that reason, the potency of DTG against the IN mutant is also reported in the table that reports the FCs of the new compounds relative to the FCs for DTG (Table S1B). Differences in potencies among the INSTIs were considered significant if the calculated *p* values were <0.05.

There was no measurable loss of potency (compared to WT) when **6u**, **6v**, and **6w** were tested against the RAL-resistant mutants Y143R and N155H (Figure 2, Table S1A). Conversely, the well-known RAL-resistant double mutant G140S/Q148H caused a loss in susceptibility to all three of the new sulfonyl-containing derivatives. For the IN double mutant G140S/Q148H, the EC_50_ values for **6u** was 117.8 ± 31.3 nM (FC = 4.4) and for **6w** was 97.5 ± 16.8 nM (FC = 9.2). The antiviral potency of **6v** against the IN double mutant G140S/Q148H was 412.5 ± 119.5 nM (FC = 1.5). These antiviral data and the calculated FCs relative to DTG potencies suggest that compounds **6u**, **6v**, and **6w** would not be effective if challenged by mutants than contain the G140S/Q148H mutations (Table S1B). These antiviral data also suggest that slight modifications to the substituents of an INSTI can cause a substantial loss of potency against both WT HIV-1 and the well-known RAL-resistant IN mutants. Additionally, consistent with the previous report detailing the importance of the amino group at the 4-position of the naphthyridine core,^26^ replacement of this amine with a hydroxyl group (**6w**) caused a large loss of potency. We previously determined the antiviral potencies of **5′g** against the RAL-resistant mutants.^26^ Compound **5j**, which has a hydroxymethyl group at the 5-position, represents a new group of naphthyridine-based INSTIs. Based on our assays, compound **5j** was more potent against WT HIV-1 (7.3 ± 0.6 nM, all *p* values <0.01) and the RAL-resistant mutant Y143R (2.6 ± 0.4 nM, *p* values ≤0.01) than were the new the sulfonyl-containing derivatives. Compound **5j** was superior to **6u** and **6v** against N155H (*p* values <0.01). The RAL-resistant mutant Y143R was susceptible to **5j** (2.6 ± 0.4 nM; FC = 0.4), whereas there was a small reduction in potency, which was considered significant when compared to its efficacy against WT HIV-1, against the IN mutant N155H (14.9 ± 3.6 nM; FC = 2.0, *p* values <0.05) and the IN double mutant G140S/Q148H (14.7 ± 1.1 nM; FC = 2.0 and *p* values <0.001); these FCs are comparable to the FCs for DTG (Table S1B).

### Antiviral Activities of DTG, **4d**, **4f**, **5′g**, and the New Compounds against Mutations in the β4-α2 Loop of HIV-1 IN

**Figure 3:**
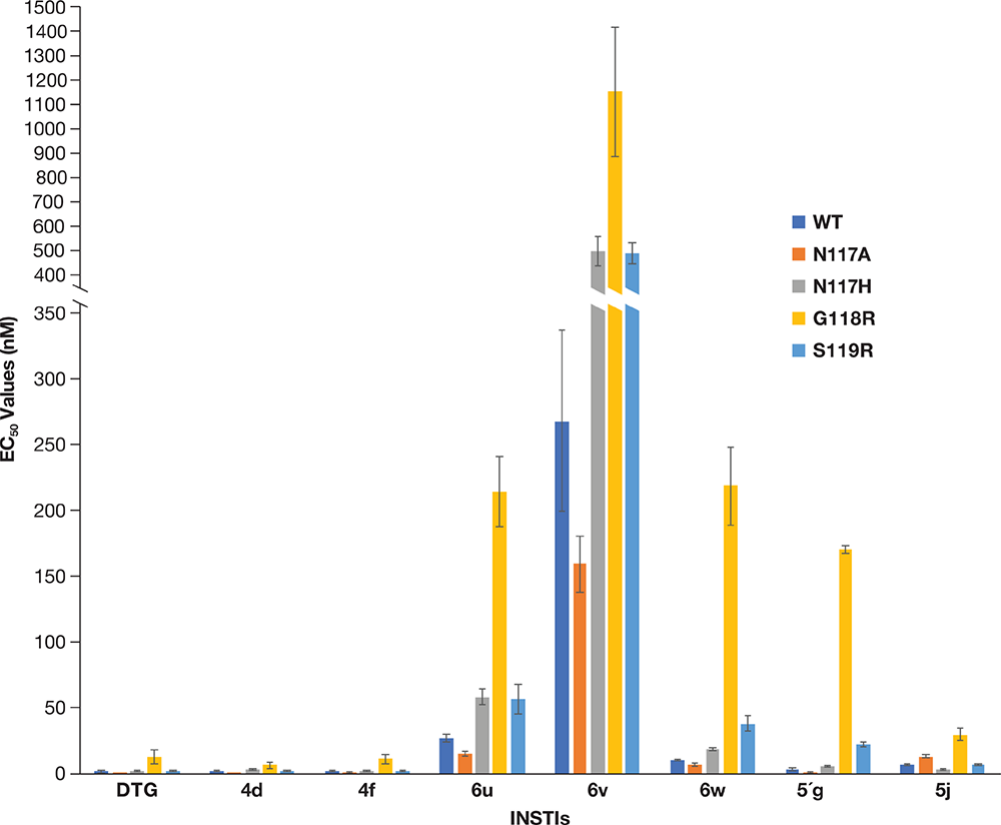
Antiviral activities of DTG, **4d**, **4f**, **5′g**, and the new compounds against mutations in the connecting loop (β4-α2) near the active site. The EC_50_ values were determined using a vector that carries the IN mutant in a single round infection assay. The *y*-axis was broken between 350 and 400 nM to better demonstrate the higher EC_50_ values. Error bars represent the standard deviations of independent experiments, *n* = 4, performed in triplicate. EC_50_ values shown in the graph have a maximum value of 1500 nM.

To better understand the interactions of the new sulfonyl-containing derivatives and the 5-substituted naphthyridine analogue **5j** with the HIV-1 intasome, we examined how mutations in and around the active site affected the ability of the compounds to inhibit the replication of WT and mutant viruses. We were particularly interested in mutations at positions that are known to affect the susceptibility of HIV-1 to DTG. These include G118 and S119, which are located on the β4-α2 loop in close proximity to the HIV-1 IN active site (Figure S1). DTG directly contacts G118,^21^ and it is not surprising that the G118R mutation was selected by DTG in cells infected in culture.^13^ Resistance mutations at position G118 appear to play an important role in determining which substituents in our compounds effectively mimic the binding of viral and/or host DNA. We previously showed that G118R causes a decrease in susceptibility to DTG (13.0 ± 5.0 nM) and **4f** (11.4 ± 3.5 nM), while causing a very minor decrease in the potency of **4d** (6.4 ± 2.5 nM).^20,25^ To understand the effect of amino acid substitutions at positions G118 and S119, we tested the antiviral potencies of **5′g**, **5j**, **6u**, **6v**, and **6w** against the known INSTI-resistant mutants G118R and S119R (Figure 3; Table S2A). We also tested additional variants with mutations at position N117 (Figure 3; Table S2A). N117 helps define the substrate envelope and can interact with some of the modifications of the 6-position in the naphthyridine scaffold of our compounds. The IN mutant G118R caused a decrease in susceptibility to all compounds used in this study. The potencies of **6u**, **6v**, and **5j** were less affected by the G118R mutation with EC_50_ values of 214.4 ± 26.3 nM (FC = 7.9), 1147.5 ± 269.7 nM (FC = 4.3), and 30.0 ± 5.0 nM (FC = 4.1), respectively. However, their potencies were all much lower that DTG against G118R; DTG was >16.5 more potent than the new compounds (Table S2B). A substantial decrease in susceptibility was seen for **6w** (218.9 ± 29.8 nM; FC = 20.7) and **5′g** (170.9 ± 3.2 nM, FC = 45). Conversely, S119R caused a minor drop in susceptibility when tested against **5′g** (22.3 ± 1.7 nM; FC = 5.9), **6u** (56.4 ± 11.0 nM; FC = 2.1), **6v** (489.9 ± 43.1 nM; FC = 1.8), and **6w** (38.2 ± 6.0 nM; FC = 3.6). However, when taking in consideration the EC_50_ values for the new compounds relative to DTG against S119R, these compounds were all much less effective (Table S2B). **5j** retained considerable potency against S119R (7.0 ± 0.2 nM, FC = 1.0). The compounds were more potent in terms of their ability to inhibit the N117A mutant when compared to WT HIV-1: DTG (0.5 nM ± 0.1, FC = 0.3), **4d** (0.6 nM ± 0.1, FC = 0.3), **5′g** (1.2 ± 0.2 nM, FC = 0.3), **6u** (15.0 nM ± 1.7, FC = 0.6), **4f** (0.8 nM ± 0.2, FC = 0.4), **6v** (159.5 nM ± 21.1, FC = 0.6), and **6w** (6.4 nM ± 1.3, FC = 0.6). The IN mutant N117A caused a minor loss in susceptibility to **5j** (13.4 ± 1.5 nM, FC = 1.8). The N117H mutant was susceptible to DTG (2.4 nM ± 0.4, FC = 1.5), **4d** (3.0 nM ± 0.5, FC = 1.3), **4f** (2.7 nM ± 0.2, FC = 1.4), **5′g** (5.6 ± 0.9 nM, FC = 1.5), and **5j** (3.4 ± 0.4 nM, FC = 0.5) and showed small decreases in susceptibility to **6u** (58.3 ± 5.9 nM, FC = 2.2), **6v** (497.4 nM ± 60.2 nM, FC = 1.9), and **6w** (19.0 ± 1.4 nM, FC = 1.8). Among the new INSTIs, compound **5j** was superior against three out of four IN mutants: N117H (*p* values <0.001), G118R (*p* values <0.001), and S119R (*p* values <0.01). The antiviral data suggest that in designing future INSTIs, it will be more important to consider potential interactions with G118 and with mutants that have changes at this position (see Discussion) rather than to try to exploit interactions with residues N117 and S119.

### Mutations in the β5-α3 Loop Affect the Antiviral Potencies of the New Compounds

**Figure 4:**
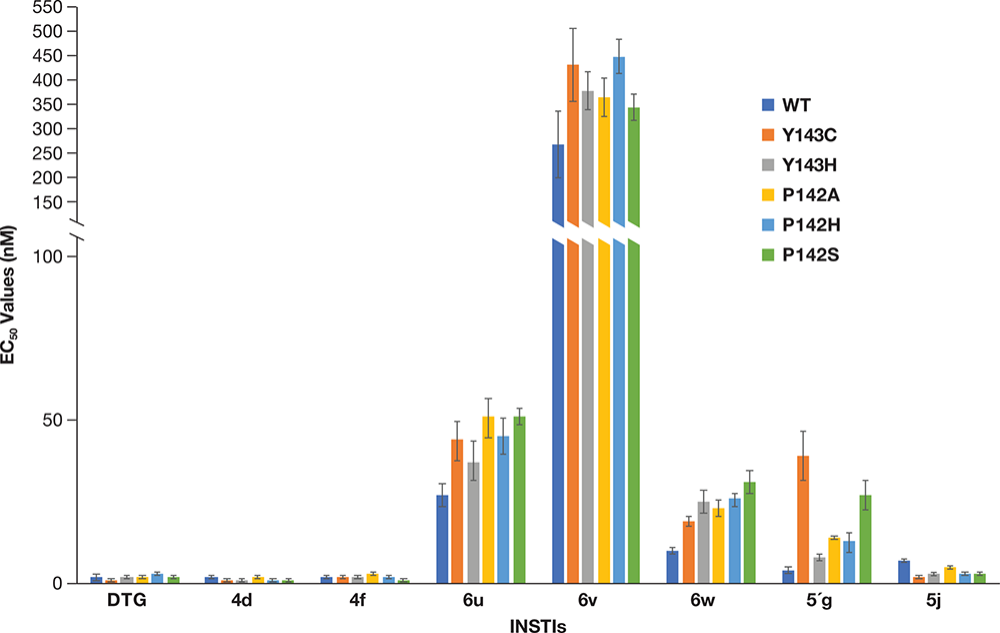
Mutations in the β5-α3 loop affect the antiviral potencies of the new compounds. The EC_50_ values were determined using a vector that carries the IN mutant in a single round infection assay. The *y*-axis was broken between 100 and 150 nM to better show the higher EC_50_ values. Error bars represent the standard deviations of independent experiments, *n* = 4, performed in triplicate. The EC_50_ values shown in the graph have a maximum value of 550 nM.

First generation INSTIs, such as RAL, select resistance mutations in the β5-α3 loop, which is adjacent to the active site of HIV IN, including Y143R and G140S/Q148H. As discussed above, there was no loss of antiviral potency for the new compounds **6u**, **6v**, **6w**, and **5j** against Y143R. However, two additional well-known INSTI-resistant mutations, Y143C and Y143H, can arise at position 143 (Figure S2); Y143 contributes to the substrate envelope. We determined the efficacies of the compounds against these INSTI-resistant mutants to see whether changes at this position affect the potencies of the compounds (Figure 4; Table S3A). DTG, **4d**, and **4f** retained potency against the additional Y143 single mutants (<3.0 nM), as did **6u** and **6w** (>18.0 nM, FCs ranging from 1.4 to 2.4). Compound **5j** showed an increase in potency against Y143C (2.4 ± 0.1 nM, FC = 0.3) and Y143H (2.6 ± 0.2 nM, FC = 0.4) when compared to WT but **5′g** lost potency against Y143C (39.3 ± 7.3 nM, FC = 10.3) and Y143H (7.9 ± 1.0 nM, FC = 2.1). This shows that, when using the naphthyridine scaffold, there are modifications that can be made around the core without compromising the susceptibility of these compounds to mutations at the Y143 position. Similar results were obtained with mutations at P142 (FCs ranged from 0.6 to 2.9 for the new compounds **6u**, **6v**, **6w**, and **5j**). However, when taking the EC_50_ values of **6u** and **6v** against the IN substitutions at positions P142 and Y143 into account, and comparing them to the EC_50_ values of DTG, these two compounds were less effective than DTG (Table S3B). The antiviral data suggest that compounds **6u**, **6w**, and **5j** are not significantly affected by the mutations we tested in the β5-α3 loop, and that, by extension, this portion of IN does not seem to contact the new compounds.

### Antiviral Potencies of the New Compounds against INs with Mutations in the C-Terminal Domain

**Figure 5:**
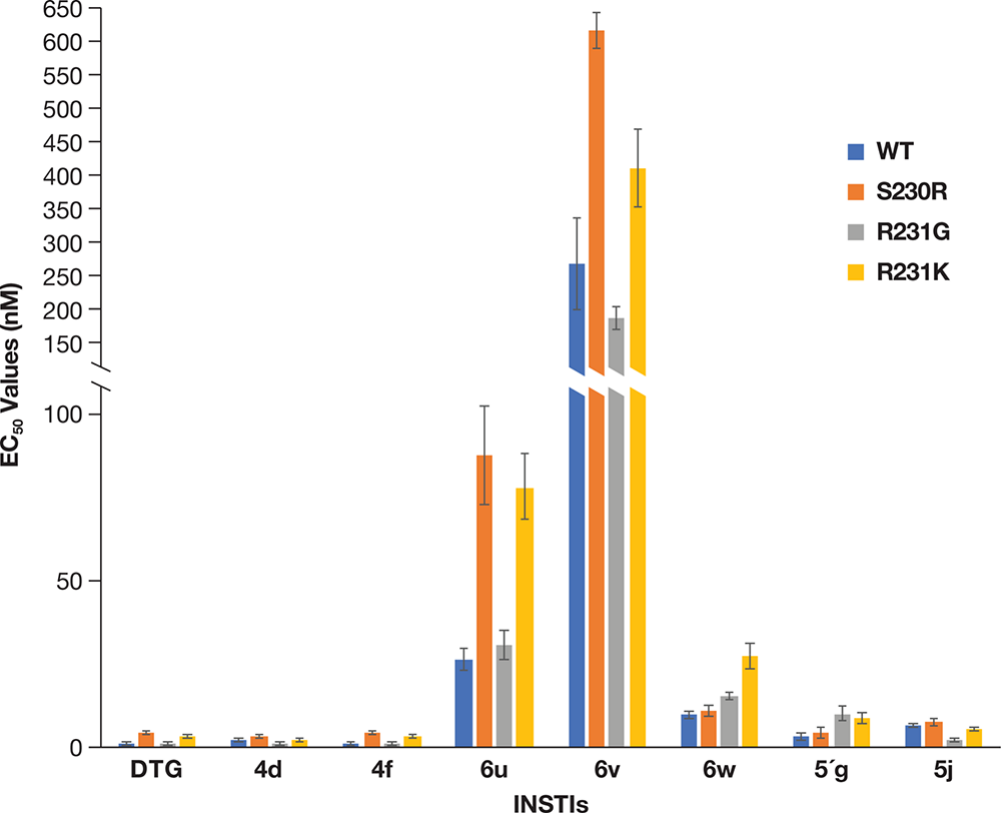
Antiviral potencies of the new compounds against IN with mutations in the C-24 terminal domain. The EC_50_ values were determined using a vector that carries the IN mutant in a single round infection assay. The *y*-axis was broken between 100 and 150 nM to better illustrate the higher EC_50_ values. Error bars represent the standard deviations of independent experiments, *n* = 4, performed in triplicate. The EC_50_ values shown in the graph have a maximum value of 650 nM.

It has been suggested that residue R231, which is in the C-terminal domain (CTD) of HIV-1 IN, may be a potential binding contact that could be exploited in the design of new INSTIs (Figure S2).^36^ There is also the possibility that mutations at this position could affect the susceptibility of IN to certain INSTIs. To explore this possibility, we made the R231G and R231K HIV IN mutants and tested them against DTG and our compounds to determine whether the potency of the compounds was affected. We also tested DTG and our compounds against the INSTI-resistant mutant S230R (Figure 5; Table S4A), which has recently been reported to be selected by DTG treatment in cultured cells.^37^ The R231G did not substantially affect the potency of DTG and most of our compounds (FCs ranging from 0.3 to 2.6), although there was a slight increase in the potency of **6v** (187.3 ± 17.0 nM, FC = 0.7). DTG, **4d**, and **4f** potently inhibited R231K (<5.0 nM, FCs = 0.9, 0.6, and 1.0, respectively). However, this mutation caused a slight decrease in potency for **6v** (411.0 ± 58.0 nM, FC = 1.5), **6u** (78.4 ± 9.9 nM, FC = 2.9), and **6w** (28.2 ± 3.9 nM, FC = 2.7). In our assays, the new IN mutant S230R, which was selected by DTG *in vitro*, caused a decrease in susceptibility to DTG (4.6 ± 0.7 nM, FC = 2.9), which is in agreement with a previous report.^37^ The S230R mutant was susceptible to **4d** (3.8 ± 0.3 nM, FC = 1.7), and **4f** (4.8 ± 0.3 nM, FC = 2.4). Compounds **6w**, **5′g**, and **5j** were the least affected by the S230R mutation with EC_50_ values of 11.2 ± 1.6, 4.5 ± 1.5, and 7.7 ± 1.0 nM, respectively, with fold changes that were not significant. This mutant caused a modest decrease in susceptibility to **6u** (88.0 ± 14.5 nM, FC = 3.3) and **6v** (616.3 ± 79.8 nM, FC = 2.3). Comparing the EC_50_ values of the compounds and DTG against these IN mutants showed that compounds **6u** and **6v** were not able to effectively inhibit these mutants (Table S4B). Despite not making a direct contact with INSTIs, S230 and R231 are within contact distances of Y143, and the S230R and R231K mutants could either make contact with INSTIs having extended substituents and/or could affect the binding of some INSTIs by interacting with Y143. Our results show that certain mutations in the CTD loop, at positions where there are differences among HIV-1, PFV, and SIV INs, can affect the potency of the compounds used in this study.

### Targeting Water Molecules at the Catalytic Core of the HIV-1 Intasome

**Figure 6:**
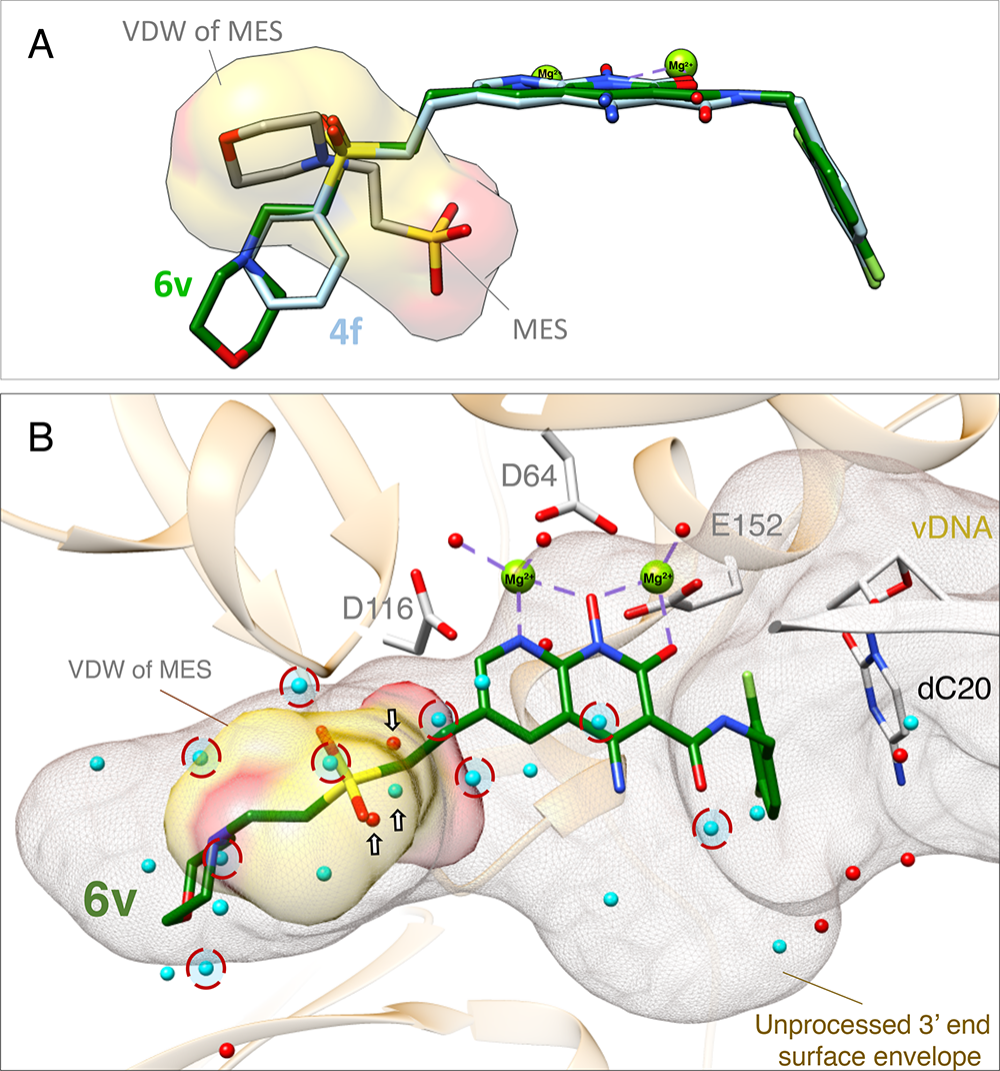
Modeling of 6v in the HIV-1 intasome. (A) Compounds **4f**, **6v**, and Buffer MES were superimposed in the active site of the HIV-1 intasome. The binding of **6v** (green) was predicted by docking it onto the structure of **4f** (silver) in the active site of the HIV-1 intasome. (B) van der Waals (VDW) surface representation of MES (yellow and red). Water molecules observed in the HIV intasome apo (PDB ID: 6PUT, cyan) and PFV-**6v** (red) structures that are within 4 Å from compound **6v** (green) are depicted. Red dashed circles indicate clashes between the waters observed in the HIV apo structure and PFV bound **6v** structure, whereas the arrows point to waters that overlap with the sulfonyl group of MES. The protein backbone (depicted in light orange), Mg^2+^ cofactors (green), the penultimate cytosine of the viral DNA (light brown), and active site DDE motif (white) are labeled. The surface envelope of the unprocessed 3′ vDNA end (brown mesh) and VDW surface representation of MES (yellow and red) are also labeled.

**Figure 7:**
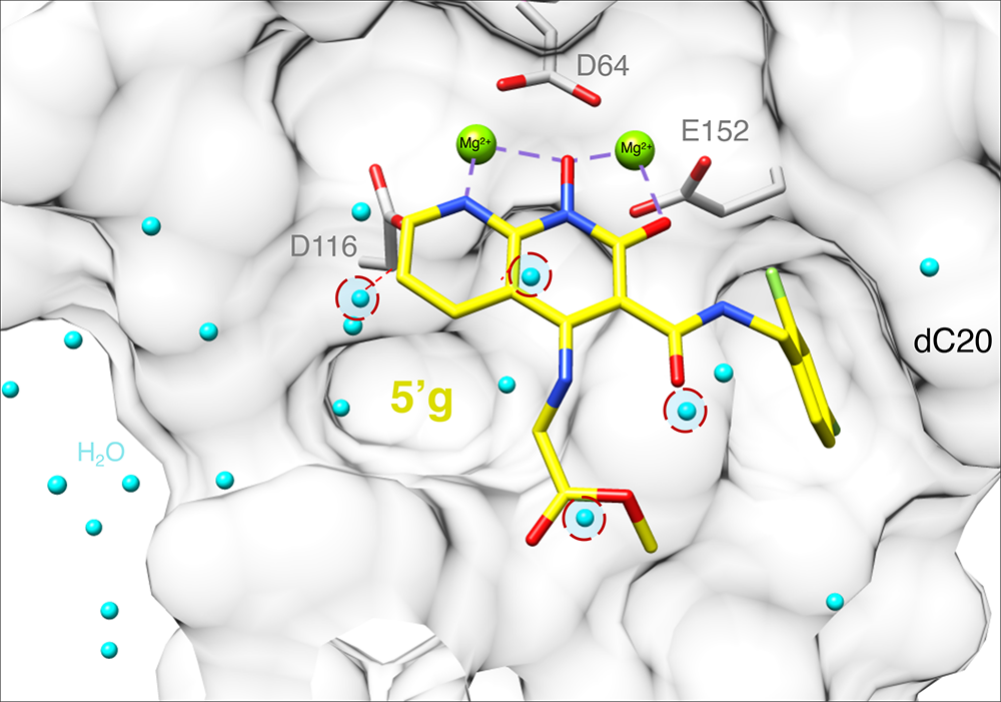
Modeling of **5′g** in the HIV-1 intasome. The binding of **5′g** (yellow) from the PFV crystal structure (PDB ID: 5MMA) was superimposed onto the structure of HIV-1 IN in complex with **4d** (PDB ID: 6PUY) represented by its surface (white density). The penultimate cytosine of the viral DNA end white density) is labeled along with the Mg^2+^ cofactors (green) and catalytic residues of the IN active site (light gray). Water molecules from HIV apo intasome within 4 Å away from **5′g** (cyan) and red dashed circles are depicted to reveal clashes between the waters of apo HIV-1 intasome structure and the binding of **5′g** into the active site of the HIV-1 intasome.

**Figure 8:**
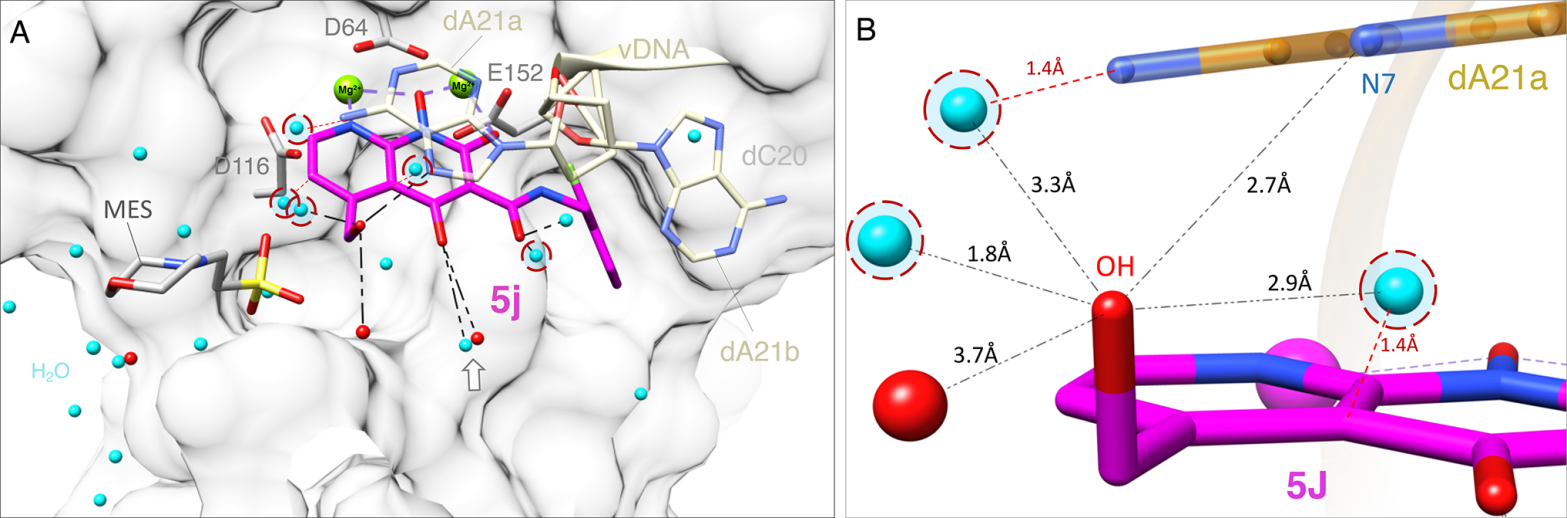
Modeling **5j** into the active site of the HIV-1 intasome. (A) **5j** (magenta) is docked onto the structure of **4d** bound to the HIV-1 intasome, which is represented by its surface (white density). Two different rotameric conformations of the terminal adenine at the end of the viral DNA (dA21a and dA21b, labeled cream) are shown, along with the penultimate cytosine of the viral DNA end (dC20, surface map and labeled in light gray), the Mg^2+^ cofactors (green), and catalytic residues of the IN active site (gray). Water molecules (cyan) that lie in close proximity to **5j** are labeled, and red dashed circles are depicted to reveal clashes between the waters of apo HIV-1 intasome and the binding of **5j** into the active site of the HIV-1 intasome. The assigned water molecules from the PFV-5j intasome structure (red) and black dashed lines indicate waters within 4 Å distance from the polar groups of **5j**. An arrow indicates a conserved water molecule between both models (PFV-5j and HIV-apo). Bound MES is also depicted (gray). (B) Cluster of potential interactions involving the hydroxyl group at 5-position of **5j** (magenta) with the terminal adenine (dA21a, gold) and water molecules from HIV-1 intasome apo (cyan) in the active site of the HIV-1 intasome. Red dashed circles are depicted to reveal clashes involving these water molecules. Water molecules from PFV-**5j** model within 4 Å from **5j** (red) are also depicted.

Recently, we determined the structures of the HIV-1 intasome with BIC, **4d**, and **4f** bound to the active site.^24^ These new structures showed that there are several ordered water molecules around the IN active site, which can potentially be exploited by rationally designed compounds (Figure S1). With this in mind, we used the intasome-bound structures of **4d** and **4f** ^24^ to model the new compounds **6v**, **5′g**, and **5j** and the apo model to explore the water network at the catalytic core (Figures 6, 7, and 8). The modification at the 6-position of **6v** (MES group) had a similar trajectory as the 6-modification of **4f**, although the modification on **6v** occupied a greater portion of the substrate envelope (Figure 6A and B). The sulfonyl group of **6v**, like the modification on **4f**, was intended to mimic the binding position of the molecule MES in the active site of the intasome; however, in the compound, it is in an inverted conformation relative to the binding of MES.^26^ When superimposed with the apo model, the bound MES group of **6v** would clash with several water molecules (Figure 6B, red dashed circles), suggesting that the binding of this compound might displace bound water molecules and potentially cause further structural rearrangements in this region. Compounds **5j** and **5′g** are compact and lack the third pharmacophore ring and/or substituents at the 6-position, but both have modifications at 4- and/or 5-position (Figure 1). Compound **5′g** has a methyl glycinate group at the 4-position that extends toward the solvent region, which could displace a distal water molecule, while the secondary amine has the potential to recoordinate a displaced water (Figure 7). **5j** has a hydroxyl group at the 4-position that could hydrogen bond with a water molecule that is consistently observed in that region (Figure 8A, arrow).^24^ The hydroxymethyl group at the 5-position could partially displace a water molecule. This, in turn, could potentially allow the compound to make a hydrogen bond with the base of nucleotide dA21, as is observed in the PFV intasome-bound crystal structure (Figure 8 and highlighted with a red circle in Figure S3). As discussed below, interaction with the vDNA could explain the ability of this compound to broadly inhibit the resistant variants used in this study.

### Single-Round Replication of the IN Mutants

Generally speaking, IN mutants have a reduced ability to replicate when compared to WT HIV-1.^19,38^ We measured the ability of the IN mutants used in this study, N117A/H, S119R, P142A/H/S, Y143C/H, S230R, and R231G/K, to replicate in a single round assay (Table S5). Although the amino acids at all of these positions are conserved among the different HIV-1 strains, they are less conserved among the other retroviral INs.^39−41^ The amino acid substitutions we made in the β4α2 loop of HIV IN caused large reductions in replication compared to WT HIV-1. The replication the N117A mutant was 10.0 ± 3.5, while the replications of N117H and S119R were 30.0 ± 3.7 and 48.4 ± 4.5, respectively. The amino acid substitutions we made in the β5α3 loop all caused reductions in viral replication in the single round infectivity assays compared to WT HIV-1; however, these reductions were not as severe as the reductions caused by the mutations in the β4α2 loop. The IN mutants P142H (73.6 ± 12.5) and P142S (83.4 ± 17.9) caused slight reductions in the single round replication assays. The reduction in the replication of the IN mutants P142A (60.2 ± 11.0), Y143C (59.4 ± 12.4), and Y143H (66.5 ± 19.9) were larger. The IN mutants, S230R and R231G, which are in the CTD, caused minor drops in replication, 71.0 ± 10.3 and 81.9 ± 19.4, respectively, whereas the replication of the R231K mutant was reduced to 51.2 ± 1.2. The data show that, of the IN mutants we tested, those in which the substitutions are in the β4α2 loop did not replicate as well in cultured cells as the mutants with substitutions at other positions in IN. This suggests that it will be important to develop inhibitors that make contacts with the β4α2 loop because it appears that mutations in this part of IN have a negative effect on the ability of the virus to replicate.

## Discussion

The second generation INSTIs, DTG and BIC, have emerged as broadly effective antiretroviral drugs. However, INSTI-experienced patients who were transferred to salvage therapies with DTG have experienced virological failure due to emergence of new mutations.^22,23^ Thus, DTG is most effective when prescribed to anti-HIV-1 therapy-naïve or HIV-1 therapy-experienced, but INSTI-naïve patients. BIC has only recently been used in the clinic and, although the preliminary data are very promising, it remains to be seen how well this drug will perform in the long term. We do know that there are mutations in IN that, in tissue culture assays, greatly reduce the susceptibility of the virus to DTG and BIC, with only modest effects on the ability of the virus to replicate.^35,38,42^ Thus, there is a need to continue to develop new and improved INSTIs that can be used to treat resistant viruses as they arise.

**Figure 9:**
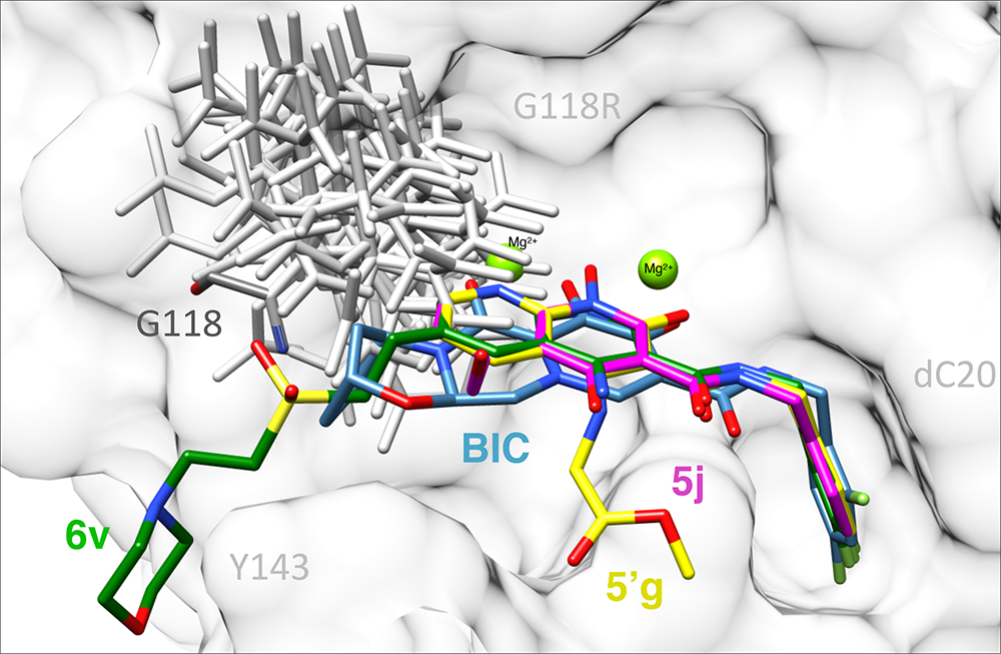
Model of the binding of INSTIs to the HIV-1 IN mutant G118R. BIC (blue), **5′g** (yellow), **5j** (magenta), and **6v** (green) are superimposed in the active site of the HIV-1 intasome. Rotamer possibilities for the mutant G118R are depicted to show the potential steric hindrance of this mutation on the binding of INSTIs. Mg^2+^ ions (green), penultimate cytosine of the viral DNA (gray), IN residues G118 and Y143 (dark and light gray, respectively), and IN mutant G118R (mild gray) are labeled.

The INSTI pharmacophore stems from the first-in-class compound, a diketo acid, and these small molecules largely share strengths as well as liabilities, which include their dependence on metal coordination.^4,21,43^ However, small changes in the structures on an INSTI have been shown to result in greatly broadened activity against clinically relevant HIV-1 IN mutants.^25,26^ For these reasons, we have focused on developing and optimizing INSTIs that are effective against the known resistant HIV-1 IN mutants. Initially using crystal structures of PFV intasomes and, more recently, cryo-EM structures of HIV-1 and SIVrcm intasomes, we have attempted to design INSTIs that make multiple contacts with the active site of HIV-1 IN. However, there are mutations in the active site, particularly G118R on the β4-α2 loop (Figure S2), that appear to interfere with the contacts made between IN and the oxazine ring of DTG or the oxazepine ring of BIC (Figure S1). The oxazine/oxazepine moieties of the second generation drugs are also referred to as a “third ring” that mediates interactions that are at least partially responsible for the success of these compounds against many drug-resistant variants.^21^ We now show that the G118R mutant reduces the potency of DTG by ∼10-fold (Figure 3; Table S2). We can begin to explain the mechanism through modeling. A change to a bulky residue, such as an arginine, would provide some steric hindrance with the third ring (Figure 9). Although a single mutation does not, by itself, profoundly affect the potency of DTG, it appears to provide a pathway for the emergence of novel resistant variants. Additional structural changes appear to propagate in the intasome beyond the immediate vicinity of the third ring. For example, the G118R mutant had a more dramatic loss in potency against compound **5′g** (∼50-fold difference between WT and G118R) and the previously studied lead compound **5′d** (16.1 ± 4.6 nM).^26^ Compound **5′g** does not contain a third ring or any pharmacophore in the vicinity of the mutation at position 118, but instead contains a methyl glycinate moiety at the 4-position. In addition, G118R caused a 4-fold reduction in potency for the small compound **5j** (Figure 3; Table S2). Addition of constituents to the 4- and 7-positions of the naphthyridine scaffold significantly reduced the antiviral potencies and increased the cytotoxicities of the compounds.^25,26,30^ However, we were able to create derivatives with modifications at the 6-position that can potently inhibit the G118R IN mutant, such as **4d** and **4f** (Figure 3; Table S2). Our results suggest that G118 helps to define the upper left periphery of the WT IN active site and that adding a bulkier side chain at position 118, typically an arginine, reduces the size of the active site (Figure 9) while potentially leading to other structural changes that can also affect smaller INSTIs that lack 4-substituents, such as **5′g** or **5j** (Figures 1, 3; Table S2). This, in turn, may reduce the binding and the potency of compounds that impinge on this portion of the active site (Figure 9).

We previously suggested that appending functionalities at the 6-position of our compounds can assist in binding efficiency by mimicking aspects of the binding of host and/or viral DNA.^25,26^ An important component of our efforts to develop more effective antiretrovirals includes challenging the compounds with HIV-1 IN mutants to understand which modifications will, and which will not, lead to broader activity. Based on the promising results that were obtained with compound **4f**, which contains an important sulfonyl modification that confers potency to the compound, and structural data obtained using PFV IN that showed a MES molecule bound to the active site and within the substrate envelope of the unprocessed 3′-end viral DNA (Figure 6), we prepared additional sulfonyl derivatives to see if we could improve the binding of the compounds, particularly to mutant forms of IN. Although **4f** has shown promising results when challenged against a variety of IN mutants, it shows susceptibility to certain mutations. Here, we investigated the effects of a sulfonyl moiety, which is key to the potency of **4f**,^25^ on compounds **6u**, **6v**, and **6w**. Our results revealed that compound **6w** had the best performance, followed by **6u**. By contrast, **6v** showed poor EC_50_ values (>250 nM) even against WT IN.

The simplest sulfonyl-containing derivative, **6u**, which has a 6-methylsulfonylethyl, showed intermediate activity when compared with the other derivatives, such as **4f** and **6v** (Figures 2–5; Tables S1–S4). The addition of a single phenyl group, which converts **6u** into **4f**, conferred substantial potency to the compound. This can be explained by the fact that the phenyl moiety of **4f** fits snugly into a cleft formed between the base of Y143 and the backbone of N117. Van der Waals interactions between the phenyl ring of **4f** and the protein cleft, and possible weak π-π stacking interactions with Y143, may explain the increase in potency. This result reinforces the importance of the phenyl ring of **4f** for its potency.

Our rationale of adding the sulfonyl group was to mimic the binding of MES to generate favorable interactions with the solvent area. However, the cryo-EM structure of HIV-1 intasome with **4f** bound and the current modeling of **6v** show that their sulfonyl groups are positioned in an opposite orientation when compared to the bound MES seen with the PFV crystal structures (Figure 6A and PDB ID: 5MMA). The potency conferred by the sulfonyl group in certain compounds, such as **4f**, can be explained by the specific interactions it makes with the local solvent environment. In contrast to **4f**, compound **6v** contains a larger 6-extension. This compound still fits well within the substrate envelope defined by the unprocessed viral DNA 3′-end (Figure 6B), but **6v** had a significantly poorer overall inhibitory profile than **4f** (Figures 1–5). Based on the crystal structure of **6v** bound to the PFV intasome (Figure S4) and the cryo-EM structure of **4f** bound to the HIV-1 intasome, it is clear that the binding modes of compounds **6v** and **4f** are very similar (Figure 6A). Therefore, the differences in potency are largely due to the differences in the 6-substituents and specifically the morpholine ring of **6v** (which is extended by two additional carbons from the sulfonyl group) and the phenyl ring of **4f**. In contrast to **6v**, which contains a hydrophilic morpholino group with two polar atoms, **4f** contains an aromatic phenyl ring and is apolar. The more extended moiety on **6v** would be expected to displace at least one additional water molecule and could displace as many as nine water molecules in total (Figure 6, red circles). Whether or not water displacement is favorable depends on numerous factors, including the hydrophobicity/hydrophilicity of the pocket, the ligand, and the resulting rearrangements and interactions in the binding pocket. Desolvation of solvent-exposed regions, such as what we report here, is less well understood, and conflicting results have been reported.^44,45^ In the current case, replacement of the phenyl ring of **4f** by the larger and more polar morpholino amine in **6v** appears to be less thermodynamically favorable, perhaps due to an unfavorable rearrangement of the hydration shell or a change in the polarity of the substituent with respect to the local environment, which may lead to loss in potency.

There is now consistent evidence, for our napthyridine compounds, that having an electron donating group, such as a primary amine, at the 4-position provides an advantage against important clinically relevant drug resistant IN mutants. Compound **4f** is identical to compound **6w**, with the exception of the substituent at the 4-position; **4f** has a 4-amino group, whereas **6w** has a 4-hydroxyl group (Figure 1). Previous studies have shown the benefits of replacing a hydroxyl group at the 4-position of naphthyridine compounds with an amino group.^26,30^ In the current work, compound **4f** consistently outperformed **6w** against all tested resistant variants (Figures 2–5, Tables S1–S4). These two compounds were also tested against the IN G140S/Q148H double mutant, which is an important combination mutant that causes virological failure with both first- and second-generation INSTIs;^46^ the mechanism of resistance for the G140S/Q148H double mutant has been recently explained.^21^ First, the introduction of histidine at position 148 displaces a key water molecule located in the secondary coordination shell of the Mg^2+^ ions bridging two of the three catalytic carboxylates (D116, E152) and Q148. Second, the interaction with S140 increases the electropositivity of H148, which is adjacent to E152. This redistributes the local charge around the Mg^2+^-ligand cluster and weakens the interaction between the drug heteroatoms and the metal ions. A similar phenomenon is thought to apply when either arginine or lysine residues are introduced at position 148 (Q148H/R/K). As a result, the G140S/Q148H/K/R double mutant confers broad cross-resistance to all INSTIs.^21^ Notably, in our current data, the most significant difference in potency between compounds **4f** and **6w** was observed when the compounds were tested against the G140S/Q148H double mutant (**6w** had a weaker efficacy against G140S/Q148H when compared to **4f**, and the difference in potencies resulted in a FC = 18.8, Table S1). In recent work, we showed that having an amino group in the 4-position has several benefits.^24^ First, it establishes an intramolecular hydrogen bond with the nearby halobenzyl amide carbonyl, which stabilizes the planar conformation of the pharmacophore. Second, and perhaps more importantly, the increased electron donating potential of the primary amine, coupled to resonance effects on the aromatic ring, strengthens the metal-ion chelation. This strengthening is expected to be particularly important in the presence of the G140S/Q148H/K/R double mutant. This result supports earlier findings with other less potent compounds. For example, we previously showed that compound **5′d**, which carries an amino group at the 4-position, outperforms compound **5′a**, which carries a hydroxyl group at the same position.^26^ Therefore, the results of the current study reinforce the impact of a hydrogen bond and provide a possible pathway for combatting the clinically problematic IN G140S/Q148H double mutant.

Compound **5j** was by far the most broadly effective of the new compounds we tested, suggesting that additional modifications to the 5-position of the naphthyridine scaffold should be explored. Modeling suggests that the OH of the 5-hydroxmethyl moiety of **5j** may interact with the adenine at the end of the viral DNA (Figure 8). If this interpretation is correct, it could be a favorable interaction, because the adenosine at this position is part of the invariant pCpA dinucleotide, which is found in all retroviral LTRs.^47−51^ As expected, substitutions at this position of the viral genome have a negative impact on viral replication.^49,51−53^ The terminal adenosine, dA21, is known to adopt multiple conformations, which appears to be dependent on several factors, one of which is the bound INSTI. Stacking interactions between the first ring of the central pharmacophore and the adenine base of dA21 should be strengthened by the hydrogen bonding interaction between the hydroxyl moiety and the N7 of adenine (Figure 8). In addition, the crystal structure of the PFV intasome with **5j** bound showed two water molecules that form hydrogen bonds with the hydroxyl moieties at the 4- and 5-positions (Figure 8A). The cryo-EM structure of HIV-1 intasome (in the absence of INSTIs) suggested that a corresponding cluster of water molecules could potentially form a similar network of hydrogen bonds (Figure 8B). Although **5j** is less potent against WT HIV-1 than **4d**, DTG, or BIC, it retains much of its potency against the mutant viruses we tested. Importantly, **5j** lacks a 6-modification; modifications at this position are important determinants for retaining antiviral efficacy against resistant mutants for our other broadly effective inhibitors. Moreover, **5j** bears a hydroxyl group at the 4-position, which as explained above, could be replaced by an amino group. Both *n*-hexanol at the 6-position and an amino group at the 4-position are present in our most potent preclinical compound **4d**. Therefore, it would be interesting to synthesize and test compounds that have various combinations of these and similar modifications. The efficacies of compounds with different combinations of specific substituents at 4-, 5-, and 6-positions will address open questions and could lead to the development of potent compounds that are capable of retaining activity against a broader range of resistant variants.

## Methods

### Cell-Based Assays

WT and mutant HIV-1 viral vectors were used in single-round infectivity assays to determine the antiviral potencies (EC_50_ values) of the compounds and the effects of the mutants on the EC_50_ values as previously described.^30,54^ Single round infectivity measurements were determined as done previously.^38^

### Vector Constructs

The vector pNLNgoMIVR-Δ*E*NV.LUC has been described previously.^26^ To produce the new IN mutants used in this study, the IN open reading frame was removed from pNLNgoMIVR-Δ*E*NV.LUC by digestion with *Kpn*I and *Sal*I, and the resulting fragment was inserted between the *Kpn*I and *Sal*I sites of pBluescript KS+. Using that construct as the wild-type template, we prepared the following HIV-1 IN mutants using the QuikChange II XL site directed mutagenesis kit (Agilent Technologies, Santa Clara, CA) protocol: N117A, N117H, P142A, P142H, P142S, Y143C, Y143H, S230R, R231G, and R231K. The following sense oligonucleotides were used with matching cognate antisense oligonucleotides (not shown) (Integrated DNA Technologies, Coralville, IA) in the mutagenesis: N117A, 5′-AAAACAGTACATACAGACGCTGGCAGCAATTTCACCAGT-3′; N117H, 5′-AAAACAGTACATACAGACCATGGCAGCAATTTCACCAGT-3′; P142A, 5′-AAGCAGGAATTTGGCATTGCCTACAATCCCCAAAGTCAA-3′; P142H, 5′-AAGCAGGAATTTGGCATTCACTACAATCCCCAAAGTCAA-3′; P142S, 5′-AAGCAGGAATTTGGCATTTCCTACAATCCCCAAAGTCAA-3′; Y143C, 5′- CAGGAATTTGGCATTCCCTGCAATCCCCAAAGTCAAGGA-3′; Y143H, 5′-CAGGAATTTGGCATTCCCCATAATCCCCAAAGTCAAGGA-3′; S230R, 5′- CGGGTTTATTACAGGGACAGAAGAGATCCAGTTTGGAAA-3′; R231G, 5′-GTTTATTACAGGGACAGCGGAGATCCAGTTTGGAAAGGA-3′; R231K, 5′-GTTTATTACAGGGACAGCAAAGATCCAGTTTGGAAAGGA-3′.

The DNA sequence of each construct was verified independently by DNA sequence determination. The mutated IN coding sequences from pBluescript KS+ were then subcloned into pNLNgoMIVR-Δ*E*nv.LUC (between the *Kpn*I and *Sal*I sites) to produce mutant HIV-1 constructs, which were also checked by DNA sequencing.

### Computer Modeling

All modeling was conducted using MOE 2019.01 02 (Chemical Computing Group, Montreal, Quebec, Canada). The sequences and structures of **4d** (PDB ID: 6PUY) and **4f** (PDB ID: 6PUZ) in the active site of the HIV-1 intasome served as the structural templates to dock **6v** and **5j**, respectively, into the active site of the HIV-1 intasome. The docking placement methodology triangle matcher, which was initially scored by London dG. Rigid receptor was used for the post refinement, and the final scoring methodology was GBVI/WSA dG.

### Synthesis

The sulfonyl-containing analogues **6u**, **6v**, and **6w** were synthesized by procedures similar to those used in the preparation of compound **4f** (Scheme S1).^25,26^ A key Heck reaction was employed by reacting bromides **7a**(25) or **7b**(25) with vinylsulfones **8a**–**c**. Coupling of bromide **7a** with commercially available methylsulfonylvinyl **8a** catalyzed by tris(dibenzylideneacetone)dipalladium(0) afforded **9a**. Coupling of bromide **7a** with freshly prepared 4-(2-(vinylsulfonyl)ethyl)morpholine **8b**afforded **9b**. Deprotection of **9a** and **9b** with TFA afforded amines **10a** and **10b**. Finally, hydrogenolytic deprotection of the *N*-benzoxyl group (H_2_, 10% Pd·C) with simultaneous reduction of the unsaturated alkenes in **10a** and **10b** gave the desired final amides **6u** and **6v**. Compound **6w** could also be prepared by coupling bromide **7b**(25) with commercially available vinylsulfonylbenzene **5c**, followed by debezylation and reduction of the resulting **9c**.

### General

^1^H and ^13^C NMR data were obtained on 400 or 500 MHz spectrometers (Varian) and are reported in ppm relative to TMS and referenced to the solvent in which the spectra were collected. Solvent was removed by rotary evaporation under reduced pressure, and anhydrous solvents were obtained commercially and used without further drying. Purification by CombiFlash silica gel chromatography was performed using EtOAc–hexanes solvent systems. Preparative high-pressure liquid chromatography (HPLC) was conducted using a Waters Prep LC4000 system having photodiode array detection and a Phenomenex C_18_ column (Cat. No. 00G-4436-P0-AX, 250 × 21.2 mm 10 μm particle size, 110 Å pore) at a flow rate of 10 mL/min. Binary solvent systems consisting of A = 0.1% aqueous TFA and B = 0.1% TFA in acetonitrile were employed with gradients as indicated. Products were obtained as amorphous solids following lyophilization. Electrospray ionization-mass spectrometric (ESI-MS) was acquired with an Agilent LC/MSD system equipped with a multimode ion source. Purities of samples subjected to biological testing were assessed using this system and shown to be ≥95%. High resolution mass spectra (HRMS) were acquired with a LTQ-Orbitrap-XL at 30K resolution by LC/MS-ESI.

### General Procedure A for the Synthesis of **9a**–**c**

A suspension of amide1-(benzyloxy)-6-bromo-*N*-(2,4-difluorobenzyl)-4-((2,4-dimethoxybenzyl)amino)-2-oxo-1,2-dihydro-1,8-naphthyridine-3-carboxamide (**7a**)^25^ or 1-(benzyloxy)-6-bromo-*N*-(2,4-difluorobenzyl)-4-hydroxy-2-oxo-1,2-dihydro-1,8-naphthyridine-3-carboxamide (**7b**)^25^ (0.6 mmol), tris(dibenzylideneacetone)dipalladium(0) (0.02 mmol), vinylsulfone (**8a**–**c**) (0.6 mmol), tri*tert*-butylphosphonium tetrafluoroborate (0.04 mmol), and *N*-cyclohexyl-*N*-methylcyclohexanamine (1.2 mmol) in dioxane (2.5 mL) in a sealed vessel charged with argon was subjected to microwave irradiation (120 °C, 12 h). The resultant mixture was cooled to RT and subjected to purification by CombiFlash to afford amides **9a**, **9b**, and **9c**.

### General Procedure B for the Synthesis of **10a** and **10b**

The 2,4-dimethoxybenzylamino-protected carboxamides **9a** and **9b** (0.25 mmol) were dissolved in DCM (2.0 mL) and treated with TFA (2.0 mL) at rt. Volatiles were removed by rotary evaporation under reduced pressure, and the resulting residues were subjected to purification by silica gel CombiFlash chromatography to afford compounds **10a** and **10b**.

### General Procedure C for the Synthesis of **6u**–**w**

Carboxamides **10a**, **10b**, or **9c** (0.1 mmol) were suspended in MeOH (10 mL) and EtOAc (3.0 mL), and Pd·C (30 mg, 10%) was added. The reaction mixture was stirred at rt under hydrogen. When consumption of starting material was completed (by TLC), the mixture was filtered and washed (MeOH), and the filtrate was concentrated to provide a yellow residue, which was taken up in DMF and subjected to HPLC purification to afford products **6u**–**w**.

#### (*E*)-1-(Benzyloxy)-*N*-(2,4-difluorobenzyl)-4-((2,4-dimethoxybenzyl)amino)-6-(2-(methylsulfonyl)vinyl)-2-oxo-1,2-dihydro-1,8-naphthyridine-3-carboxamide (**9a**)

Reaction of 1-(benzyloxy)-6-bromo-*N*-(2,4-difluorobenzyl)-4-((2,4-dimethoxybenzyl)amino)-2-oxo-1,2-dihydro-1,8-naphthyridine-3-carboxamide (**7a**)^25^ with commercially available (methylsulfonyl)ethene (**8a**) as outlined in General Procedure A provided **9a** as a yellow solid (14% yield). ^1^H NMR (500 MHz, CDCl_3_) δ 12.36 (t, *J* = 6.8 Hz, 1H), 10.71 (t, *J* = 5.7 Hz, 1H), 8.69 (d, *J* = 1.7 Hz, 1H), 8.40 (d, *J* = 1.7 Hz, 1H), 7.69 (d, *J* = 6.4 Hz, 2H), 7.50 (d, *J* = 15.5 Hz, 1H), 7.44–7.39 (m, 5H), 6.89–6.83 (m, 2H), 6.65 (d, *J* = 2.1 Hz, 1H), 6.58 (dd, *J* = 8.4, 2.2 Hz, 1H), 6.10 (d, *J* = 15.5 Hz, 1H), 5.27 (s, 2H), 4.75 (d, *J* = 6.9 Hz, 2H), 4.65 (d, *J* = 5.7 Hz, 2H), 3.89 (s, 3H), 3.87 (s, 3H), 2.94 (s, 3H).

#### (*E*)-1-(Benzyloxy)-*N*-(2,4-difluorobenzyl)-4-((2,4-dimethoxybenzyl)amino)-6-(2-((2-morpholinoethyl)sulfonyl)vinyl)-2-oxo-1,2-dihydro-1,8-naphthyridine-3-carboxamide (**9b**)

Reaction of **7a** with 4-(2-(vinylsulfonyl)ethyl)morpholine (**8b**) as outlined in General Procedure A provided **9b** as a yellow solid (60% yield). ^1^H NMR (400 MHz, CDCl_3_) δ 12.35 (t, *J* = 6.7 Hz, 1H), 10.68 (t, *J* = 5.7 Hz, 1H), 8.70 (d, *J* = 1.6 Hz, 1H), 8.37 (d, *J* = 1.6 Hz, 1H), 7.69−7.67 (m, 2H), 7.44 (d, *J* = 15.6 Hz, 1H), 7.42−7.37 (m, 5H), 6.89−6.81 (m, 2H), 6.62 (d, *J* = 2.1 Hz, 1H), 6.54 (dd, *J* = 8.4, 2.1 Hz, 1H), 6.26 (d, *J* = 15.5 Hz, 1H), 5.27 (s, 2H), 4.74 (d, *J* = 6.7 Hz, 2H), 4.64 (d, *J* = 5.6 Hz, 2H), 3.88 (s, 3H), 3.85 (s, 3H), 3.58−3.56 (m, 4H), 3.16 (t, *J* = 6.9 Hz, 2H), 2.78 (t, *J* = 6.9 Hz, 2H), 2.43−2.40 (m, 4H). ESI-MS *m*/*z*: 790.3 (MH^+^).

#### (*E*)-1-(Benzyloxy)-*N*-(2,4-difluorobenzyl)-4-hydroxy-2-oxo-6-(2-(phenylsulfonyl)vinyl)-1,2-dihydro-1,8-naphthyridine-3-carboxamide (**9c**)

Reaction of **7b**(25) with commercially available (vinylsulfonyl)benzene (**8c**) as outlined in General Procedure A provided **9c** as a white solid (18% yield). ^1^H NMR (400 MHz, CDCl_3_) δ 10.15 (t, *J* = 5.8 Hz, 1H), 8.78 (d, *J* = 2.0 Hz, 1H), 8.49 (d, *J* = 2.0 Hz, 1H), 7.92–7.90 (m, 2H), 7.69 (d, *J* = 15.5 Hz, 1H), 7.57–7.52 (m, 5H), 7.34–7.28 (m, 5H), 6.97 (d, *J* = 15.5 Hz, 1H), 6.82–6.75 (m, 2H), 5.18 (s, 2H), 4.58 (d, *J* = 5.9 Hz, 2H). ESI-MS *m*/*z*: 604.1 (MH^+^).

#### (*E*)-4-Amino-1-(benzyloxy)-*N*-(2,4-difluorobenzyl)-6-(2-(methylsulfonyl)vinyl)-2-oxo-1,2-dihydro-1,8-naphthyridine-3-carboxamide (**10a**)

Treatment of **9a** as outlined in General Procedure B provided **10a** as a yellow solid (88% yield). ^1^H NMR (500 MHz, DMSO-*d*_6_) δ 10.78 (bs, 1H), 10.42 (t, *J* = 5.8 Hz, 1H), 9.17 (s, 1H), 9.08 (d, *J* = 1.4 Hz, 1H), 7.67–7.64 (m, 3H), 7.57 (d, *J* = 15.6 Hz, 1H), 7.48–7.40 (m, 4H), 7.28–7.248 (m, 1H), 7.09 (td, *J* = 8.5, 1.8 Hz, 1H), 5.17 (s, 2H), 4.55 (d, *J* = 5.7 Hz, 2H), 3.18 (s, 3H). ESI-MS: *m*/*z*: 541.1 (MH^+^).

#### (*E*)-4-Amino-1-(benzyloxy)-*N*-(2,4-difluorobenzyl)-6-(2-((2-morpholinoethyl)sulfonyl)vinyl)-2-oxo-1,2-dihydro-1,8-naphthyridine-3-carboxamide (**10b**)

Treatment of **9b** as outlined in General Procedure B provided **10b** a yellow solid (87% yield). ^1^H NMR (400 MHz, CDCl_3_) δ 10.87 (bs, 1H), 10.38 (t, *J* = 5.7 Hz, 1H), 8.63 (s, 1H), 8.59 (s, 1H), 7.54–7.50 (m, 3H), 7.33–7.26 (m, 4H), 7.19 (s, 1H), 7.13 (d, *J* = 15.4 Hz, 1H), 6.77–6.70 (m, 2H), 5.13 (s, 2H), 4.52 (d, *J* = 5.6 Hz, 2H), 3.74–3.70 (m, 4H), 3.50–3.44 (m, 2H), 3.21–3.19 (m, 1H), 2.88–2.85 (m, 4H). ESI-MS *m*/*z*: 640.2 (MH^+^).

#### 4-Amino-*N*-(2,4-difluorobenzyl)-1-hydroxy-6-(2-(methylsulfonyl)ethyl)-2-oxo-1,2-dihydro-1,8-naphthyridine-3-carboxamide (**6u**)

Treatment of **10a** as outlined in General Procedure C and purification by preparative HPLC (linear gradient of 30% B to 50% B over 30 min; retention time = 20.5 min) provided **6u** as white solid (25% yield). ^1^H NMR (400 MHz, DMSO-*d*_6_) δ 10.67 (t, *J* = 5.8 Hz, 1H), 8.68 (d, *J* = 1.8 Hz, 1H), 8.65 (s, 1H), 7.43 (dd, *J* = 15.4, 8.6 Hz, 1H), 7.28–7.22 (m, 1H), 7.10–7.05 (m, 1H), 4.53 (d, *J* = 5.6 Hz, 2H), 3.54 (dd, *J* = 9.6, 6.7 Hz, 2H), 3.16 (dd, *J* = 9.6, 6.6 Hz, 2H), 3.04 (s, 3H). ESI-MS *m*/*z*: 453.1 (MH^+^). HRMS calcd for C_19_H_19_F_2_N_4_O_5_S (MH^+^): 453.1039; Found: 453.1040.

#### 4-Amino-*N*-(2,4-difluorobenzyl)-1-hydroxy-6-(2-((2-morpholinoethyl)sulfonyl)ethyl)-2-oxo-1,2-dihydro-1,8-naphthyridine-3-carboxamide (**6v**)

Treatment of **10b** as outlined in General Procedure C purification by preparative HPLC (linear gradient of 20% B to 50% B over 30 min; retention time = 19.5 min) provided **6v** a as white solid (14% yield). ^1^H NMR (400 MHz, DMSO-*d*_6_) δ 8.66 (d, *J* = 2.1 Hz, 1H), 8.59 (d, *J* = 2.0 Hz, 1H), 7.42 (dd, *J* = 15.6, 8.5 Hz, 1H), 7.22 (dd, *J* = 14.7, 5.1 Hz, 1H), 7.06 (t, *J* = 8.6 Hz, 1H), 4.50 (s, 2H), 3.76–3.71 (m, 4H), 3.41–3.39 (m, 4H), 3.19–3.15 (m, 4H), 3.11–3.08 (m, 4H). ESI-MS *m*/*z*: 552.2 (MH^+^). HRMS calcd for C_24_H_29_F_2_N_5_O_6_S (MH^+^): 552.1723; Found: 552.1713.

#### *N*-(2,4-Difluorobenzyl)-1,4-dihydroxy-2-oxo-6-(2-(phenylsulfonyl)ethyl)-1,2-dihydro-1,8-naphthyridine-3-carboxamide (**6w**)

Treatment of **9c** as outlined in General Procedure C and purification by preparative HPLC (with a linear gradient of 45% B to 60% B over 30 min; retention time = 23.4 min) provided **6w** as a white solid (39% yield). ^1^H NMR (400 MHz, DMSO-*d*_6_) δ10.43 (t, *J* = 5.8 Hz, 1H), 8.63 (d, *J* = 2.2 Hz, 1H), 8.23 (d, *J* = 2.2 Hz, 1H), 7.85–7.81 (m, 2H), 7.67–7.61 (m, 1H), 7.59–7.53 (m, 2H), 7.41 (dd, *J* = 15.3, 8.7 Hz, 1H), 7.24–7.18 (m, 1H), 7.02 (td, *J* = 8.6, 2.3 Hz, 1H), 4.56 (d, *J* = 6.0 Hz, 2H), 3.77–3.73 (m, 2H), 3.03–2.99 (m, 2H). ESI-MS *m*/*z*: 516.1 (MH^+^).

#### *N*-(2,4-Difluorobenzyl)-1,4-dihydroxy-5-(hydroxymethyl)-2-oxo-1,2-dihydro-1,8-naphthyridine-3-carboxamide (**5j**)

**5j** was prepared as a white solid.^56^^1^H NMR (500 MHz, DMSO-*d*_6_) δ 10.91 (brs, 1H), 10.63 (brs, 1H), 8.75 (d, *J* = 5.0 Hz, 1H), 7.65 (d, *J* = 4.9 Hz, 1H), 7.46 (dd, *J* = 15.3, 8.5 Hz, 1H), 7.28–7.24 (m, 1H), 7.08 (t, *J* = 8.6 Hz, 1H), 5.09 (s, 2H), 4.62 (d, *J* = 6.0 Hz, 2H). ESI-MS *m*/*z*: 378.10 (MH^+^), 400.00 (MNa^+^). HRMS calcd for C_17_H_14_F_2_N_3_O_5_ (MH^+^), 378.0896; Found: 378.0902.

### X-ray Crystallography

PFV intasome crystals were grown as previously described,^20,57^ soaked in the presence of either 0.5–1 mM **5j** or **6v** in cryoprotection solution prior to snap freezing in liquid nitrogen. X-ray diffraction data were collected on beamline I03 at the Diamond Light Source (Oxfordshire, UK). Data were processed using XDS^58^ or Dials^59^ and Aimless^60^ via Xia2,^61^ the structure was determined via rigid-body refinement of a ligand- and solvent-free model generated from PDB ID 4BDZ; the compounds **5j** and **6v** were fitted into resulting positive *F*_o_–*F*_c_ difference maps. The models were built in Coot,^62^ refined using Phenix version dev-3900,^63^ and validated using MolProbity.^64^ Relevant data collection and refinement statistics are given in Table S5 and the structure coordinates and reflection files have been deposited with the PDB under the accession codes 7ADU and 7ADV.

## Supporting Information Available

Scheme S1, preparation of sulfonyl-containing analogues **6u**–**w**; Figure S1, cryo-EM structure showing **4d**, DTG, and BIC bound to the HIV-1 intasome; Figure S2, positions of amino acid substitutions in proximity to the HIV-1 IN active site; Figure S3, crystal structure of **5j** in the active site of the PFV intasome; Figure S4, crystal structure of **6v** in the active site of the PFV intasome; Table S1A, antiviral activities of the new compounds against RAL-resistant mutants; Table S1B, antiviral activities of the new compounds against RAL-resistant mutants; Table S2A, antiviral activities of DTG, **4d**, **4f**, **5′g**, and the new compounds against mutation in the connecting loop (β4-α2) near the active site; Table S2B, antiviral activities of DTG, **4d**, **4f**, **5′g**, and the new compounds against mutation in the connecting loop (β4-α2) near the active site; Table S3A, mutations in the β5-α3 loop affect the antiviral potencies of the new compounds; Table S3B, mutations in the β5-α3 loop affect the antiviral potencies of the new compounds; Table S4A, antiviral potencies of the new compounds against IN with mutations in the C-terminal domain; Table S4B, antiviral potencies of the new compounds against IN with mutations in the C-terminal domain; Table S5, replication of IN mutants using a single round infectivity assay; Table S6, data collection, phase, and refinement statistics; Table S6, data collection and refinement statistics (PDF)
