# Supplementary material for: HIV-1 Integrase Inhibitors with Modifications That Affect Their Potencies against Drug Resistant Integrase Mutants
Source: ACS Infect Dis. 2021 Mar 9;7(6):1469–82. doi: 10.1021/acsinfecdis.0c00819 (PMC8205226; doi:10.1021/acsinfecdis.0c00819)
Supplement: Supplementary file 1 — id0c00819_si_001.pdf [file id0c00819_si_001.pdf]

Supporting Information

HIV-1 integrase inhibitors with modifications that affect their potencies against drug resistant integrase mutants

Steven J. Smith[1], Xue Zhi Zhao[2], Dario Oliveira Passos[3], Valerie E. Pye[4], Peter Cherepanov[4,5], Dmitry Lyumkis[3,6], Terrence R. Burke, Jr.[2], and Stephen H. Hughes[1*]

[1] HIV Dynamics and Replication Program, Center for Cancer Research, National Cancer Institute, Frederick, MD, 21702, USA

[2] Chemical Biology Laboratory, Center for Cancer Research, National Cancer Institute, Frederick, MD, 21702, USA

[3] Laboratory of Genetics, The Salk Institute for Biological Studies, La Jolla, CA 92037, USA

[4] Chromatin Structure and Mobile DNA Laboratory, The Francis Crick Institute, London NW1 1AT, UK

[5] Imperial College London, St Mary's Hospital, Department of Infectious Disease, Section of Virology, Norfolk Place, London, W2 1PG, UK

[6] Department of Structural and Computational Biology, The Scripps Research Institute, La Jolla, CA 92037, USA

* Corresponding author

Pages: 11

Scheme: 1

Figures: S1-S4

Tables: Table S1-S6

Supporting Information

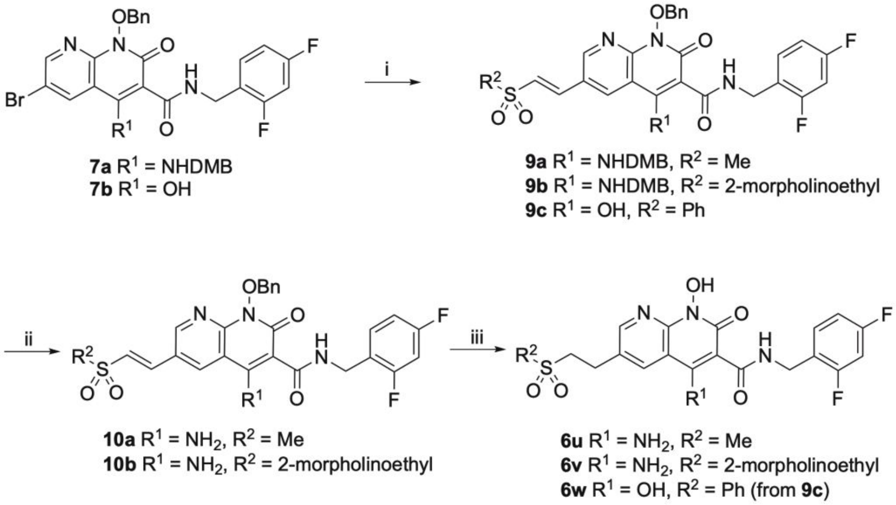

Scheme S1. Preparation of sulfonyl-containing analogues **6(u-w)**. *Reagents and conditions:* i) vinylsulfones [$CH_3SO_2CH{=}CH_2$ (**8a**), 2-morpholine-$CH_2CH_2SO_2CH{=}CH_2$ (**8b**), or $PhSO_2CH{=}CH_2$ (**8c**)], $Pd_2(dba)_3$, ${}^tBu_3P\text{-}BF_4$, ${}^cHex_2NMe$; ii) TFA, DCM; iii) H_2, 10% Pd/C, MeOH.

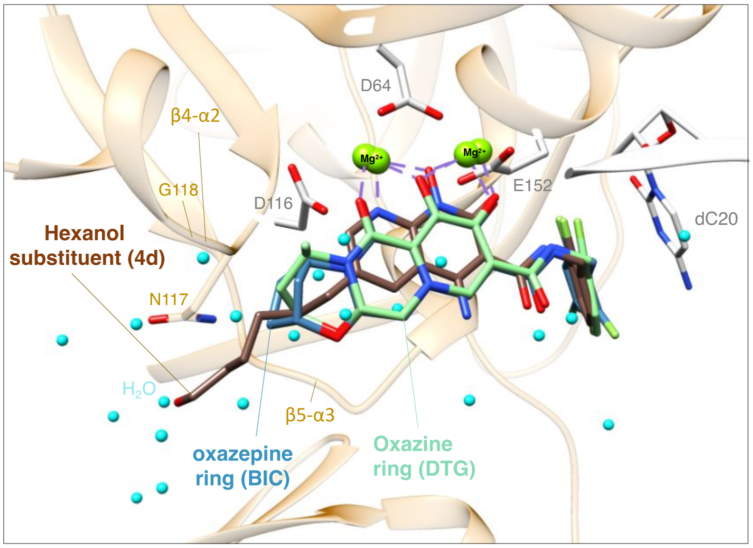

Figure S1. Cryo-EM structure showing 4d, DTG, and BIC bound to the HIV-1 intasome. 4d (6PUY, dark brown), DTG (docked, light green) and BIC (6PUW, steel blue) are shown bound in the active site of the HIV-1 intasome. Observed water molecules in and around the IN active site from the HIV intasome apo structure are also shown (cyan). The penultimate cytosine of the incoming viral DNA end is labeled (dC20, depicted in white) as are the Mg^{2+} cofactors (green).The loops involved in interactions with INSTIs (β4-α2 and β5-α3) and catalytic residues (light gray) of the IN active site are labeled. The nucleotide base of the dA21 that stacks with the INSTIs is hidden. The surface representation of the HIV-1 IN is light orange.

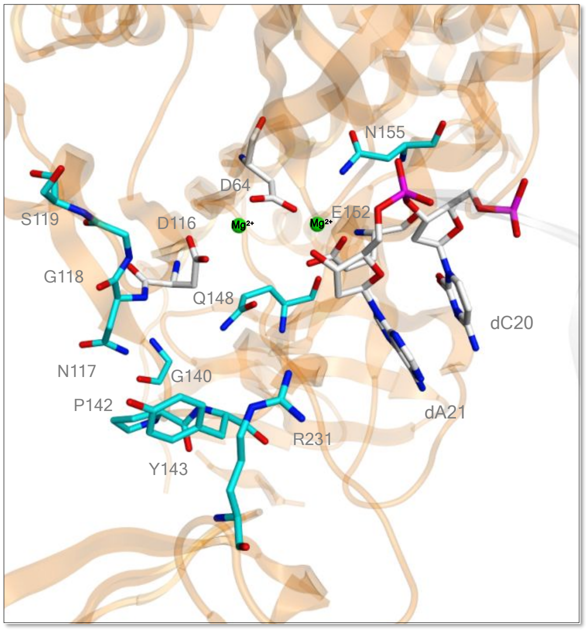

Figure S2. Positions of amino acid substitutions in proximity to the HIV-1 IN active site. The locations of the positions in proximity to the IN active site that undergo amino acid substitutions for INSTI resistance are shown in the apo-form of the HIV-1 intasome; modified from (24). The HIV-1 IN active site catalytic residues are labeled and highlighted in white, while the Mg^{2+} ions (green), protein backbone (orange), viral DNA (shown in white), and the positions that undergo amino acid substitution and are associated with INSTI resistance (shown in cyan) are labeled.

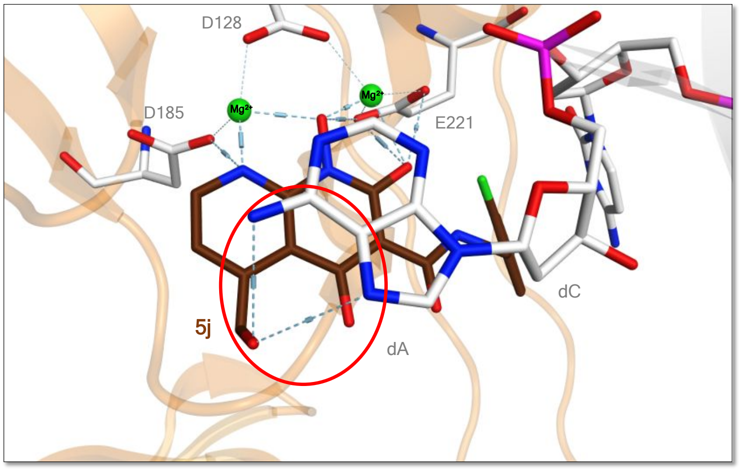

Figure S3. Crystal structure of 5j in the active site of the PFV intasome. The interaction of **5j** (brown) with the active site (DDE motif shown in white) of the PFV intasome (protein backbone in orange and viral DNA in white) are shown. The Mg^{2+} ions (shown in green) interact with the chelating motif of **5j**. The benzyl moiety of **5j** hydrophobically stacks with the penultimate cytosine near the 3' end of the viral DNA. The hydroxymethyl modification of the 5- position of the naphthyridine scaffold makes hydrogen bond interactions (blue dashed lines) with the terminal adenine (highlighted by red circle).

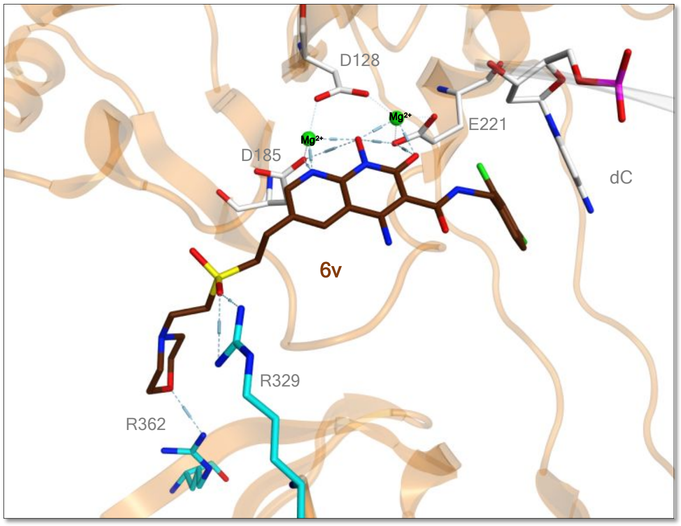

Figure S4. Crystal structure of 6v in the active site of the PFV intasome. The interactions of **6v** (brown) in the active site (DDE motif shown in white) of the PFV intasome (protein backbone in orange and viral DNA depicted in white) are shown. The Mg^{2+} ions (shown in green) interact with the chelating motif of **6v**. The benzyl moiety of **6v** hydrophobically stacks with the penultimate cytosine near the 3' end of the viral DNA. The morpholinoethylsulfonyl derivative of **6v** extends away from the PFV IN active site towards the CTD. The sulfonyl oxygens (red) of **6v** interact with R329 through hydrogen bonding interactions (blue dashed lines) and the backbone of G187 (not shown). The oxygen (red) in the morpholino ring forms a hydrogen bond (blue dashed line) with R362. Terminal adenine is hidden for clarity.

Table S1A. Antiviral Activities of the new compounds against RAL-resistant mutants. The EC_{50} values and the standard deviation (n=4) of DTG, **4d**, **4f**, **5'g** and the new compounds **6u**, **6v**, **6w**, and **5j** against RAL-resistant mutants tested are shown.

Table S1B. Antiviral activities of the new compounds against RAL-resistant mutants. The ratio of FCs, which were based on the EC_{50} values of our compounds against the IN mutants relative to WT were compared to the FCs for DTG. The FC for DTG is given before the colon and the FCs for our compounds are after the colon. In addition, the ratios of the EC_{50} values for our compounds, relative to the EC_{50} values for DTG, were determined and are shown.

Table S1A

INSTIs	EC_{50} values WT	EC_{50} values Y143R (FC)	EC_{50} values N155H (FC)	EC_{50} values G140S/Q148H (FC)
DTG	1.6 ± 0.9 nM	4.3 ± 1.2 nM (2.7)	3.6 ± 1.3 nM (2.3)	5.8 ± 0.5 nM (3.6)
4d	2.3 ± 0.6 nM	2.1 ± 1.4 nM (0.9)	2.7 ± 1.0 nM (1.2)	9.4 ± 3.6 nM (4.1)
4f	2.0 ± 0.1 nM	0.6 ± 0.1 nM (0.3)	2.1 ± 1.2 nM (1.1)	5.2 ± 0.3 nM (2.6)
6u	27.0 ± 3.2 nM	27.5 ± 0.4 nM (1.0)	32.8 ± 6.0 nM (1.2)	117.8 ± 31.3 nM (4.4)
6v	267.9 ± 68.8 nM	108.6 ± 16.2 nM (0.4)	139.7 ± 4.9 nM (0.5)	412.5 ± 119.5 nM (1.5)
6w	10.6 ± 1.0 nM	7.2 ± 1.7 nM (0.7)	14.1 ± 4.3 nM (1.3)	97.5 ± 16.8 nM (9.2)
5'g	3.8 ± 1.2 nM	4.6 ± 2.2 nM (1.2)	19.0 ± 6.9 nM (5.0)	35.5 ± 15.9 nM (9.3)
5j	7.3 ± 0.6 nM	2.6 ± 0.4 nM (0.4)	14.9 ± 3.6 nM (2.0)	14.7 ± 1.1 nM (2.0)

Table S1B

INSTIs	EC_{50} values WT	Ratios of the FCs (DTG vs. Our Compounds), and the ratios of the EC_{50}s (for DTG vs. Our Compounds) against Y143R	Ratios of the FCs (DTG vs. Our Compounds), and the ratios of the EC_{50}s (for DTG vs. Our Compounds) against N155H	Ratios of the FCs (DTG vs. Our Compounds), and the ratios of the EC_{50}s (for DTG vs. Our Compounds) against G140S/Q148H
DTG	1.6 ± 0.9 nM	4.3 ± 1.2 nM (2.7)	3.6 ± 1.3 nM (2.3)	5.8 ± 0.5 nM (3.6)
4d	2.3 ± 0.6 nM	(2.7) : (0.9); (0.5)	(2.3) : (1.2); (0.8)	(3.6) : (4.1); (1.6)
4f	2.0 ± 0.1 nM	(2.7) : (0.3); (0.1)	(2.3) : (1.1); (0.6)	(3.6) : (2.6); (0.9)
6u	27.0 ± 3.2 nM	(2.7) : (1.0); (6.4)	(2.3) : (1.2); (9.1)	(3.6) : (4.4); (20.3)
6v	267.9 ± 68.8 nM	(2.7) : (0.4); (25.3)	(2.3) : (0.5); (38.8)	(3.6) : (1.5); (71.1)
6w	10.6 ± 1.0 nM	(2.7) : (0.7); (1.7)	(2.3) : (1.3); (3.9)	(3.6) : (9.2); (16.8)
5'g	3.8 ± 1.2 nM	(2.7) : (1.2); (1.1)	(2.3) : (5.0); (5.3)	(3.6) : (9.3); (6.1)
5j	7.3 ± 0.6 nM	(2.7) : (0.4); (0.6)	(2.3) : (2.0); (4.1)	(3.6) : (2.0); (2.5)

Table S2A. Antiviral activities of DTG, 4d, 4f, 5'g, and the new compounds against mutation in the connecting loop (β4-α2) near the active site. The EC_{50} values and the standard deviation (n=4) of DTG, **4d**, **4f**, **5'g** and the new compounds **6u**, **6v**, **6w**, and **5j** against the IN mutants tested are shown.

Table S2B. Antiviral Activities of DTG, 4d, 4f, 5'g, and the new compounds against mutation in the connecting loop (β4-α2) near the active site. The ratio of FCs, which were based on the EC50 values of our compounds against the IN mutants relative to WT were compared to the FCs for DTG. The FC for DTG is given before the colon and the FCs for our compounds are after the colon. In addition, the ratios of the EC50 values for our compounds, relative to the EC50 values for DTG, were determined and are shown.

Table S2A

INSTIs	EC_{50} values WT	EC_{50} values N117A (FC)	EC_{50} values N117H (FC)	EC_{50} values G118R (FC)	EC_{50} values S119R (FC)
**DTG**	1.6 ± 0.9 nM	0.5 ± 0.1 nM (0.3)	2.4 ± 0.4 nM (1.5)	13.0 ± 5.0 nM (8.1)	2.3 ± 0.6 nM (1.4)
**4d**	2.3 ± 0.6 nM	0.6 ± 0.1 nM (0.3)	3.0 ± 0.5 nM (1.3)	6.4 ± 2.5 nM (2.8)	2.1 ± 0.1 nM (0.9)
**4f**	2.0 ± 0.1 nM	0.8 ± 0.2 nM (0.4)	2.7 ± 0.2 nM (1.4)	11.4 ± 3.5 nM (5.7)	2.6 ± 0.4 nM (1.3)
**6u**	27.0 ± 3.2 nM	15.0 ± 1.7 nM (0.6)	58.3 ± 5.9 nM (2.2)	214.4 ± 26.3 nM (7.9)	56.4 ± 11.0 nM (2.1)
**6v**	267.9 ± 68.8 nM	159.5 ± 21.1 nM (0.6)	497.4 ± 60.2 nM (1.9)	1147.5 ± 269.7 nM (4.3)	489.9 ± 43.1 nM (1.8)
**6w**	10.6 ± 1.0 nM	6.4 ± 1.3 nM (0.6)	19.0 ± 1.4 nM (1.8)	218.9 ± 29.8 nM (20.7)	38.2 ± 6.0 nM (3.6)
**5'g**	3.8 ± 1.2 nM	1.2 ± 0.2 nM (0.3)	5.6 ± 0.9 nM (1.5)	170.9 ± 3.2 nM (45.0)	22.3 ± 1.7 nM (5.9)
**5j**	7.3 ± 0.6 nM	13.4 ± 1.5 nM (1.8)	3.4 ± 0.4 nM (0.5)	30.0 ± 5.0 nM (4.1)	7.0 ± 0.2 nM (1.0)

Table S2B

INSTIs	EC_{50} values WT	Ratios of the FCs (DTG vs. Our Compounds), and the ratios of the EC_{50}s (for DTG vs. Our Compounds) against N117A	Ratios of the FCs (DTG vs. Our Compounds), and the ratios of the EC_{50}s (for DTG vs. Our Compounds) against N117H	Ratios of the FCs (DTG vs. Our Compounds), and the ratios of the EC_{50}s (for DTG vs. Our Compounds) against G118R	Ratios of the FCs (DTG vs. Our Compounds), and the ratios of the EC_{50}s (for DTG vs. Our Compounds) against S119R
**DTG**	1.6 ± 0.9 nM	0.5 ± 0.1 nM (0.3)	2.4 ± 0.4 nM (1.5)	13.0 ± 5.0 nM (8.1)	2.3 ± 0.6 nM (1.4)
**4d**	2.3 ± 0.6 nM	(0.3) : (0.3); (1.2)	(1.5) : (1.3); (1.3)	(8.1) : (2.8); (0.5)	(1.4) : (0.9); (0.9)
**4f**	2.0 ± 0.1 nM	(0.3) : (0.4); (0.8)	(1.5) : (1.4); (1.1)	(8.1) : (5.7); (0.9)	(1.4) : (1.3); (1.1)
**6u**	27.0 ± 3.2 nM	(0.3) : (0.6); (30.0)	(1.5) : (2.2); (24.3)	(8.1) : (7.9); (16.5)	(1.4) : (2.1); (24.5)
**6v**	267.9 ± 68.8 nM	(0.3) : (0.6); (319.0)	(1.5) : (1.9); (207.3)	(8.1) : (4.3); (88.3)	(1.4) : (1.8); (213.0)
**6w**	10.6 ± 1.0 nM	(0.3) : (0.6); (12.8)	(1.5) : (1.8); (7.9)	(8.1) : (20.7); (16.8)	(1.4) : (3.6); (16.6)
**5'g**	3.8 ± 1.2 nM	(0.3) : (0.3); (2.4)	(1.5) : (1.5); (2.3)	(8.1) : (45.0); (13.1)	(1.4) : (5.9); (9.7)
**5j**	7.3 ± 0.6 nM	(0.3) : (1.8); (26.8)	(1.5) : (0.5); (1.4)	(8.1) : (4.1); (2.3)	(1.4) : (1.0); (3.0)

Table S3A. Mutations in the β5-α3 Loop affect the antiviral potencies of the new compounds. The EC_{50} values and the standard deviation (n=4) of DTG, **4d**, **4f**, **5'g** and the new compounds **6u**, **6v**, **6w**, and **5j** against the IN mutants tested are shown.

Table S3B. Mutations in the β5-α3 Loop affect the antiviral potencies of the new compounds. The ratio of FCs, which were based on the EC50 values of our compounds against the IN mutants relative to WT were compared to the FCs for DTG. The FC for DTG is given before the colon and the FCs for our compounds are after the colon. In addition, the ratios of the EC50 values for our compounds, relative to the EC50 values for DTG, were determined and are shown.

Table S3A

INSTIs	EC_{50} values WT	EC_{50} values Y143C (FC)	EC_{50} values Y143H (FC)	EC_{50} values P142A (FC)	EC_{50} values P142H (FC)	EC_{50} values P142S (FC)
**DTG**	1.6 ± 0.9 nM	1.4 ± 0.5 nM (0.9)	2.2 ± 0.2 nM (1.4)	2.5 ± 0.5 nM (1.6)	2.8 ± 0.7 nM (1.8)	2.2 ± 0.1 nM (1.4)
**4d**	2.3 ± 0.6 nM	1.2 ± 0.1 nM (0.5)	1.5 ± 0.1 nM (0.7)	2.1 ± 0.3 nM (0.9)	1.5 ± 0.1 nM (0.7)	1.2 ± 0.2 nM (0.5)
**4f**	2.0 ± 0.1 nM	1.7 ± 0.4 nM (0.9)	2.3 ± 0.1 nM (1.2)	2.8 ± 0.1 nM (1.4)	1.7 ± 0.2 nM (0.9)	1.5 ± 0.3 nM (0.8)
**6u**	27.0 ± 3.2 nM	43.9 ± 5.9 nM (1.6)	37.4 ± 6.0 nM (1.4)	50.8 ± 6.4 nM (1.9)	45.1 ± 5.2 nM (1.7)	51.5 ± 2.4 nM (1.9)
**6v**	267.9 ± 68.8 nM	431.8 ± 75.3 nM (1.6)	378.3 ± 38.9 nM (1.4)	364.7 ± 39.4 nM (1.4)	447.7 ± 35.8 nM (1.7)	344.1 ± 26.9 nM (1.3)
**6w**	10.6 ± 1.0 nM	18.8 ± 1.4 nM (1.8)	25.1 ± 3.6 nM (2.4)	22.8 ± 2.5 nM (2.2)	25.7 ± 2.0 nM (2.4)	31.2 ± 3.7 nM (2.9)
**5'g**	3.8 ± 1.2 nM	39.3 ± 7.3 nM (10.3)	7.9 ± 1.0 nM (2.1)	14.1 ± 0.3 nM (3.7)	12.8 ± 2.9 nM (3.4)	27.2 ± 4.4 nM (7.2)
**5j**	7.3 ± 0.6 nM	2.4 ± 0.1 nM (0.3)	2.6 ± 0.2 nM (0.4)	4.7 ± 0.1 nM (0.6)	3.5 ± 0.1 nM (0.5)	3.5 ± 0.5 nM (0.5)

Table S3B

INSTI	EC_{50} values WT	Ratios of the FCs (DTG vs. Our Compounds), and the ratios of the EC_{50}s (for DTG vs. Our Compounds) against Y143C	Ratios of the FCs (DTG vs. Our Compounds), and the ratios of the EC_{50}s (for DTG vs. Our Compounds) against Y143H	Ratios of the FCs (DTG vs. Our Compounds), and the ratios of the EC_{50}s (for DTG vs. Our Compounds) against P142A	Ratios of the FCs (DTG vs. Our Compounds), and the ratios of the EC_{50}s (for DTG vs. Our Compounds) against P142H	Ratios of the FCs (DTG vs. Our Compounds), and the ratios of the EC_{50}s (for DTG vs. Our Compounds) against P142S
**DTG**	1.6 ± 0.9 nM	1.4 ± 0.5 nM (0.9)	2.2 ± 0.2 nM (1.4)	2.5 ± 0.5 nM (1.6)	2.8 ± 0.7 nM (1.8)	2.2 ± 0.1 nM (1.4)
**4d**	2.3 ± 0.6 nM	(0.9) : (0.5); (0.9)	(1.4) : (0.7); (0.8)	(1.6) : (0.9); (0.8)	(1.8) : (0.7); (0.5)	(1.4) : (0.5); (0.5)
**4f**	2.0 ± 0.1 nM	(0.9) : (0.9); (1.2)	(1.4) : (1.2); (1.0)	(1.6) : (1.4); (1.1)	(1.8) : (0.9); (0.6)	(1.4) : (0.8); (0.7)
**6u**	27.0 ± 3.2 nM	(0.9) : (1.6); (31.4)	(1.4) : (1.4); (17.0)	(1.6) : (1.9); (20.3)	(1.8) : (1.7); (16.1)	(1.4) : (1.9); (23.4)
**6v**	267.9 ± 68.8 nM	(0.9) : (1.6); (308.4)	(1.4) : (1.4); (172.0)	(1.6) : (1.4); (145.9)	(1.8) : (1.7); (159.9)	(1.4) : (1.3); (156.4)
**6w**	10.6 ± 1.0 nM	(0.9) : (1.8); (13.4)	(1.4) : (2.4); (11.4)	(1.6) : (2.2); (9.1)	(1.8) : (2.4); (9.2)	(1.4) : (2.9); (14.2)
**5'g**	3.8 ± 1.2 nM	(0.9) : (10.3); (28.1)	(1.4) : (2.1); (3.6)	(1.6) : (3.7); (5.6)	(1.8) : (3.4); (4.6)	(1.4) : (7.2); (12.4)
**5j**	7.3 ± 0.6 nM	(0.9) : (0.3); (1.7)	(1.4) : (0.4); (1.2)	(1.6) : (0.6); (1.9)	(1.8) : (0.5); (1.3)	(1.4) : (0.5); (1.6)

Table S4A. Antiviral potencies of the new compounds against IN with mutations in the C-terminal domain. The EC_{50} values and the standard deviation (n=4) of DTG, **4d**, **4f**, **5'g** and the new compounds **6u**, **6v**, **6w**, and **5j** against the IN mutants tested are shown.

Table S4B. Antiviral potencies of the new compounds against IN with mutations in the C-terminal domain. The ratio of FCs, which were based on the EC50 values of our compounds against the IN mutants relative to WT were compared to the FCs for DTG. The FC for DTG is given before the colon and the FCs for our compounds are after the colon. In addition, the ratios of the EC50 values for our compounds, relative to the EC50 values for DTG, were determined and are shown.

Table S4A

INSTI	EC_{50} values WT	EC_{50} values S230R (FC)	EC_{50} values R231G (FC)	EC_{50} values R231K (FC)
**DTG**	1.6 ± 0.9 nM	4.6 ± 0.7 nM (2.9)	1.4 ± 0.2 nM (0.9)	4.2 ± 0.2 nM (2.6)
**4d**	2.3 ± 0.6 nM	3.8 ± 0.3 nM (1.7)	1.3 ± 0.2 nM (0.6)	2.4 ± 0.3 nM (1.0)
**4f**	2.0 ± 0.1 nM	4.8 ± 0.3 nM (2.4)	2.0 ± 0.2 nM (1.0)	3.3 ± 0.2 nM (1.7)
**6u**	27.0 ± 3.2 nM	88.0 ± 14.5 nM (3.3)	31.5 ± 4.0 nM (1.2)	78.4 ± 9.9 nM (2.9)
**6v**	267.9 ± 68.8 nM	616.3 ± 79.8 nM (2.3)	187.3 ± 17.0 nM (0.7)	411.0 ± 58.0 nM (1.5)
**6w**	10.6 ± 1.0 nM	11.2 ± 1.6 nM (1.1)	15.2 ± 1.4 nM (1.4)	28.2 ± 3.9 nM (2.7)
**5'g**	3.8 ± 1.2 nM	4.5 ± 1.5 nM (1.2)	9.7 ± 2.4 nM (2.6)	9.2 ± 2.1 nM (2.4)
**5j**	7.3 ± 0.6 nM	7.7 ± 1.0 nM (1.1)	2.5 ± 0.6 nM (0.3)	5.9 ± 0.2 nM (0.8)

Table S4B

INSTI	EC_{50} values WT	Ratios of the FCs (DTG vs. Our Compounds), and the ratios of the EC_{50}s (for DTG vs. Our Compounds) against S230R	Ratios of the FCs (DTG vs. Our Compounds), and the ratios of the EC_{50}s (for DTG vs. Our Compounds) against R231G	Ratios of the FCs (DTG vs. Our Compounds), and the ratios of the EC_{50}s (for DTG vs. Our Compounds) against R231K
**DTG**	1.6 ± 0.9 nM	4.6 ± 0.7 nM (2.9)	1.4 ± 0.2 nM (0.9)	4.2 ± 0.2 nM (2.6)
**4d**	2.3 ± 0.6 nM	(2.9) : (1.7); (0.8)	(0.9) : (0.6); (0.9)	(2.6) : (1.0); (0.6)
**4f**	2.0 ± 0.1 nM	(2.9) : (2.4); (1.0)	(0.9) : (1.0); (1.4)	(2.6) : (1.7); (0.8)
**6u**	27.0 ± 3.2 nM	(2.9) : (3.3); (19.1)	(0.9) : (1.2); (22.5)	(2.6) : (2.9); (18.7)
**6v**	267.9 ± 68.8 nM	(2.9) : (2.3); (134.0)	(0.9) : (0.7); (133.8)	(2.6) : (1.5); (97.9)
**6w**	10.6 ± 1.0 nM	(2.9) : (1.1); (2.4)	(0.9) : (1.4); (10.9)	(2.6) : (2.7); (6.7)
**5'g**	3.8 ± 1.2 nM	(2.9) : (1.2); (1.0)	(0.9) : (2.6); (6.9)	(2.6) : (2.4); (2.2)
**5j**	7.3 ± 0.6 nM	(2.9) : (1.1); (1.7)	(0.9) : (0.3); (1.8)	(2.6) : (0.8); (1.4)

Table S5. Replication of IN mutants using a single round infectivity assay. The replication of the IN mutants used in this study were measured, in a single-round infection assay, using the vectors carrying the appropriate mutations. The luciferase activity was set to 100, and the replication of the mutant vectors (adjusted for amount of p24/Gag used in the assay) was measured and compared to WT. The error bars represent standard deviations of independent experiments, n=4, done in triplicate.

Table S5

HIV-1 IN mutant	Single Round Infectivity (luc)
WT	100
N117A	10.0 ± 3.5
N117H	30.0 ± 3.7
S119R	48.4 ± 4.5
P142A	60.2 ± 11.0
P142H	73.6 ± 12.5
P142S	83.4± 17.9
Y143C	59.4 ± 12.4
Y143H	66.5 ± 9.9
S230R	71.0 ± 10.3
R231G	81.9 ± 19.4
R231K	51.2 ± 12.2

Table S6. Data collection, phase, and refinement statistics. The data collection, phasing, and refinement statistics of the crystal structures **5j** and **6v** in the active site of the PFV intasome are shown.

Table S6. Data collection and refinement statistics.

Data Collection	6v (XZ447)	5j (XZ440)
Wavelength (Å)	0.97957	0.97957
Space Group	$P4_12_12$	$P4_12_12$
Unit cell		
a, b, c (Å)	158.61, 158.61,	159.47, 159.47,
α,β,γ (°)	90, 90, 90	90, 90, 90
No. crystals used	1	1
Resolution (Å)	70.93 -2.65 (2.74-2.65)	97.64-2.62 (2.66-2.62)
Measured	533,393	675,863
Unique	45,870(4,449)	48,012 (4,625)
Completeness (%)	99.6 (98.1)	99.4 (98.5)
Multiplicity	11.6 (11.0)	14.0 (14.5)
$<I/\sigma(I)>$	20.8 (1.5)	11.1 (1.1)
R_{merge}	0.081 (1.7)	0.167 (2.2)
R_{pim} (all I^+ and I^-)	0.027 (0.55)	0.064 (0.857)
**Refinement Statistics**		
Resolution Range	50.157 -2.65 (2.74-2.65)	71.32-2.62 (2.71-2.62)
No. of Reflections:		
Work Set	45,869 (4,449)	48,012 (4,625)
Free	2,302 (239)	2,411 (250)
R_{work}/R_{free}	0.1959/0.2192	0.202/0.2313
No. of Atoms:		
Total	5,458	5,449
Protein/DNA	5,135	5,131
Ligand	163	133
Solvent	160	185
R.m.s.d. Bonds	0.003	0.002
R.m.s.d. Angles	0.52	0.50
Average B factor	73.35	62.97
Most Favored	97.61	97.06
Disallowed (%)	0.18	0.00

[a] Values in parentheses correspond to the highest resolution bin.
[b] Analyzed using Molprobity (http://molprobity.biochem.duke.edu/).
